# Drug Shortages Prior to and During the COVID-19 Pandemic

**DOI:** 10.1001/jamanetworkopen.2024.4246

**Published:** 2024-04-05

**Authors:** Katherine Callaway Kim, Scott D. Rothenberger, Mina Tadrous, Inmaculada Hernandez, Walid F. Gellad, Joshua W. Devine, Tina B. Hershey, Lisa M. Maillart, Katie J. Suda

**Affiliations:** 1 Department of Medicine, Division of General Internal Medicine, University of Pittsburgh School of Medicine, Pittsburgh, Pennsylvania; 2 Department of Health Policy and Management, University of Pittsburgh School of Public Health, Pittsburgh, Pennsylvania; 3 Leslie Dan Faculty of Pharmacy, University of Toronto, Toronto, Ontario, Canada; 4 Women’s College Research Institute, Toronto, Ontario, Canada; 5 School of Pharmacy and Pharmaceutical Sciences, University of California San Diego, La Jolla; 6 Center of Health Equity Research and Promotion, VA Pittsburgh Healthcare System, Pittsburgh, Pennsylvania; 7 Des Moines University, Department of Public Health, Des Moines, Iowa; 8 Department of Industrial Engineering, University of Pittsburgh Swanson School of Engineering, Pittsburgh, Pennsylvania

## Abstract

**Importance:**

Drug shortages are a chronic and worsening issue that compromises patient safety. Despite the destabilizing impact of the COVID-19 pandemic on pharmaceutical production, it remains unclear whether issues affecting the drug supply chain were more likely to result in meaningful shortages during the pandemic.

**Objective:**

To estimate the proportion of supply chain issue reports associated with drug shortages overall and with the COVID-19 pandemic.

**Design, Setting, and Participants:**

This longitudinal cross-sectional study used data from the IQVIA Multinational Integrated Data Analysis database, comprising more than 85% of drug purchases by US pharmacies from wholesalers and manufacturers, from 2017 to 2021. Data were analyzed from January to May 2023.

**Exposure:**

Presence of a supply chain issue report to the US Food and Drug Administration or the American Society of Health-Systems Pharmacists (ASHP).

**Main Outcomes and Measures:**

The main outcome was drug shortage, defined as at least 33% decrease in units purchased within 6 months of a supply chain issue report. Random-effects logistic regression models compared the marginal odds of shortages for drugs with vs without reports. Interaction terms assessed heterogeneity prior to vs during the COVID-19 pandemic and by drug characteristics (formulation, age, essential medicine status, clinician- vs self-administered, sales volume, and number of manufacturers).

**Results:**

A total of 571 drugs exposed to 731 supply chain issue reports were matched to 7296 comparison medications with no reports. After adjusting for drug characteristics, 13.7% (95% CI, 10.4%-17.8%) of supply chain issue reports were associated with subsequent drug shortages vs 4.1% (95% CI, 3.6%-4.8%) of comparators (marginal odds ratio [mOR], 3.7 [95% CI, 2.6-5.1]). Shortages increased among both drugs with and without reports in February to April 2020 (34.2% of drugs with supply chain issue reports and 9.5% of comparison drugs; mOR, 4.9 [95% CI, 2.1-11.6]), and then decreased after May 2020 (9.8% of drugs with reports and 3.6% of comparison drugs; mOR, 2.9 [95% CI, 1.6-5.3]). Significant associations were identified by formulation (parenteral mOR, 1.9 [95% CI, 1.1-3.2] vs oral mOR, 5.4 [95% CI, 3.3-8.8]; *P* for interaction = .008), WHO essential medicine status (essential mOR, 2.2 [95% CI, 1.3-5.2] vs nonessential mOR, 4.6 [95% CI, 3.2-6.7]; *P* = .02), and for brand-name vs generic status (brand-name mOR, 8.1 [95% CI, 4.0-16.0] vs generic mOR, 2.4 [95% CI, 1.7-3.6]; *P* = .002).

**Conclusions and Relevance:**

In this national cross-sectional study, supply chain issues associated with drug shortages increased at the beginning of the COVID-19 pandemic. Ongoing policy work is needed to protect US drug supplies from future shocks and to prioritize clinically valuable drugs at greatest shortage risk.

## Key Points

**Question:**

What was the incidence of drug shortages in the US between 2017 and 2021?

**Findings:**

In this cross-sectional study, a total of 571 drugs exposed to 731 supply chain issue reports were matched to 7296 comparison medications with no reports from 2017 to 2021. One in 7 reports (14%) were associated with drug shortages (decreases in sales of ≥33%); supply disruptions increased for drugs with and without reports at the start of the COVID-19 pandemic and returned to prepandemic levels after May 2020.

**Meaning:**

These findings suggest that ongoing policy work is needed to protect US drug supplies from future supply shocks.

## Teaser

This cross-sectional study examines the proportion of supply chain issue reports associated with drug shortages overall and during the COVID-19 pandemic.

## Introduction

Drug shortages have reached record highs.^1^ The US Food and Drug Administration (FDA) defines a shortage as “a period of time when the demand…for the drug…exceeds the supply.”^2^ Shortages have been associated with missed or delayed dosages,^3,4^ medication errors,^5,6^ increased spending,^7,8^ and death.^9^ Shortages are caused by underreimbursement,^10^ quality concerns,^11^ and an overreliance on single-source production.^12^

Since 2012, companies must report to the FDA known issues affecting the supply chain which could result in shortages (hereafter, *supply chain issue reports*).^2^ These reports are also tracked by the American Society of Health-Systems Pharmacists (ASHP).^13^ Examples of supply chain issue reports include quality concerns (eg, microbial contamination) and unanticipated events that may impact production (eg, natural disasters).^14^ The FDA also publishes recalls and discontinuations. The COVID-19 pandemic was associated with record numbers of supply chain issue reports.^1,15^ Despite these known risks, it is unknown how often supply chain issues were associated with subsequent shortages, ie, meaningful decreases in the national supply of a medication.

As the public health emergency ends, there is an imperative need for evidence regarding its impact on the drug supply chain to inform policies^16,17,18,19,20^ that protect patients from future, unanticipated shocks.^21^ The primary objective of our study was to quantify the proportion of all supply chain issue reports that were associated with drug shortages from 2017 to 2021. Our study adds to existing literature by comparing shortages during vs prior to the COVID-19 pandemic, and by drug characteristics. Our results provide evidence to inform policies to manage upstream events before they impact patients’ access to life-saving treatments.

## Methods

We conducted a population-based, longitudinal cross-sectional study of US prescription drug purchases from January 2017 to December 2021. Using public information from the FDA and ASHP, we identified medications with supply chain issue reports and matched them to drugs without reports. We selected supply chain characteristics based on a conceptual framework and compared the odds of shortages for each group. The University of Pittsburgh Institutional Review Board approved the study as not human participants research; therefore, the requirements for approval and informed consent were waived. We followed the Strengthening the Reporting of Observational Studies in Epidemiology (STROBE) reporting guideline.

### Conceptual Model

Drug manufacturers are legally required to report known supply chain issues which could result in shortages (eg, inability to obtain raw ingredients) to the FDA; they report voluntarily to ASHP. However, supply chain reports may be incomplete indicators for meaningful decreases in national supplies (ie, shortages). As represented in eFigure 1 in Supplement 1, protective shields in supply chains,^22,23^ including opening more production lines, importing drugs from other countries, lengthening expiration dates,^14,22^ or using residual supplies,^22^ may address issues prior to the depletion of supplies held by manufacturers, wholesalers, and pharmacies. Therefore, not all reported issues necessarily result in drug shortages.

Given these complexities and the known limitations of reporting, the purpose of our study was to quantify the proportion of supply chain issue reports that were associated with subsequent population-level shortages, both prior to and during the COVID-19 pandemic (eFigure 1 in Supplement 1). Because there is no legal standard for meaningful decreases in supply, we defined shortages as decreases in purchased units of at least 33% within 6 months after the issuance of a new supply chain issue report. This threshold was chosen based on previous studies^24^ and is consistent with the FDA and ASHP’s definitions of shortages, ie, significant decreases in national supply^2^ with the greatest potential to impact patient care.^25^ We accounted for non–shortage-related supply chain factors by matching drugs exposed to reports to unexposed drugs without reports. We then compared subsequent odds of shortages (ie, ≥33% decreases in supply) for each group.

### Data Sources and Study Population

Our study population comprised a longitudinal cross-section of drugs purchased in the US from 2017 to 2021 in IQVIA’s Multinational Integrated Data Analysis (MIDAS) database. MIDAS comprises 85% of retail and 97% of nonretail purchases by pharmacies from wholesalers and manufacturers, based on unit volume.^26^ Data are reported in standardized units, defined as 1 pill, capsule, or vial or 5 mL oral liquid. We conducted our analyses on the drug formulation level. We excluded products with incomplete data, radiopharmaceuticals, unapproved products, allergens, antidotes, and over-the-counter products that are not fully captured in MIDAS. We also excluded topicals, since these may reflect different dosages that are difficult to compare with systemic formulations (eTable 1 in Supplement 1).

Our exposed group comprised drugs with incident supply chain issue reports issued from January 2017 to September 2021 (3 months prior to data end). For each report, we defined the index date as the initial posting date by the FDA or ASHP. This included issues reported as being likely to result in shortages,^2,13,27,28^ FDA recalls,^29^ and discontinuations. Reports for the same drug-form were considered the same episode if they occurred within 90 days (eFigure 2 in Supplement 1). We conducted sensitivity analyses excluding recalls and discontinuations which may be indefinite.^30^

We performed exposure density sampling (EDS) to match each drug with a supply chain issue report (ie, exposed) with up to 10 comparison drugs with no reports at the time of exposure (ie, nonexposed).^31,32^ EDS is akin to sampling techniques for nested case-control studies,^32,33,34^ with the exception that matching is performed at the time of an exposure instead of the time of an outcome. This approach mitigated bias due to seasonality. When appropriately combined with multivariable adjustment for confounders, EDS leads to unbiased results.^32^ We did not match on baseline purchasing trends, since many reports were precipitated by abnormal demand increases.^35^ Drugs could appear more than once in either group. Drugs with reports could serve as at-risk comparators during any month without an active supply issue report.^33^

### Outcome Measures of Interest

Motivated by FDA’s definition^2^ and based on previous studies,^24^ we defined meaningful shortages as at least 33% decreases in purchased units within 6 months after an incident supply chain issue report, compared with 3 months prior (eFigure 3 in Supplement 1). This decrease is greater than what would be expected based on seasonal trends. We conducted secondary analyses of severe shortages, defined as decreases of at least 66%, as in previous literature.^24^

### Statistical Analyses

We used random-effects logistic regression models followed by marginal estimation to compare the odds of shortages for drugs with vs without supply chain issue reports. We included a random intercept for each matched set to account for potential dependency induced by matching. Our main estimates were the mean marginal odds of shortages for drugs with vs without reports.^34,35^ We estimated absolute incidence by comparing the estimated probabilities of shortages in each group. A type 1 error rate of .05 was assumed, all hypothesis tests were 2-sided, and no adjustments were made for multiplicity. Analyses were conducted from January to May 2023 in SAS version 9.4 (SAS Institute), Stata version 16.1 (StataCorp), and RStudio version 4.1 (R Project for Statistical Computing).

#### Heterogeneity in Shortages Before COVID-19 vs During the COVID-19 Pandemic

To determine the association of the COVID-19 pandemic with US drug supply shortages, we included interaction terms to assess differential changes in the odds of shortages for drugs with vs without reports before the pandemic (January 2017 to January 2020), in the first few months after the World Health Organization (WHO) declared COVID-19 an emergency and when stockpiling occurred^36^ (February 2020 to April 2020), and in the first pandemic year (May 2020 to September 2021). We grouped issues based on their issuance date.

#### Heterogeneity by Baseline Characteristics

To assess the associations of supply chain characteristics with shortage incidence, we included interaction terms by formulation. We then used marginal estimation to compare odds of shortages for drugs with vs without reports, within each form, eg, parenteral drugs with reports vs parenteral comparators. Additional interaction terms were added for other characteristics, including years since market entry and generic availability from the FDA National Drug Code Directory (August 2022),^37^ WHO essential medicine status (June 2022),^38^ and clinician- vs self-administration, defined using J-codes.^39^ We proxied supply chain redundancy using the number of manufacturers in MIDAS in the 6 months prior to a supply chain issue report. To capture market size, we adjusted for total sales in MIDAS during the same period (eTable 2 in Supplement 1).

#### Sensitivity Analyses

We conducted sensitivity analyses to assess the influence of methodological assumptions. Because some shortages take longer to develop (eg, due to residual supply), we redefined shortages as decreases in supply within 9 months after report issuance. We assessed 5 (vs 10) matched comparisons, FDA and ASHP reports separately, and excluded recalls and discontinuations.^30^ Finally, we used nearest-neighbor propensity score methods to match drugs on month, formulation, class, clinician- vs self-administration, WHO essential-medicine status, at least 20 years since market or generic availability, at least 5 manufacturers, and at least $5 million in sales.

## Results

### Descriptive Data

**Figure 1.**
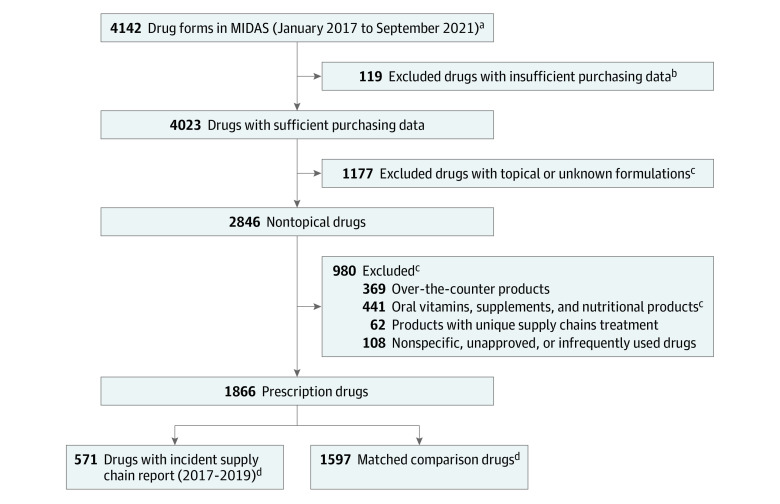
Cohort Selection Diagram MIDAS indicates Multinational Integrated Data Analysis. ^a^Drugs were defined on the Anatomical Therapeutic Class, level-3 (ATC3)-molecule-formulation level. ^b^Excludes drugs with less than 6 months of information prior to assigned index date. ^c^A list of excluded topical and nonprescription medications can be found in eTable 2 in Supplement 1. The 1597 matched comparison drugs were allowed to be selected multiple times over the study period for a total sample size of 7296 matched comparison drugs over the study period. ^d^Categories are not mutually exclusive since drugs could appear more than once in either group.

Prior to inclusion criteria, there were 4142 drugs. As shown in Figure 1, 2276 drugs met exclusion criteria, resulting in a final sample of 1866 drugs. Among these, 571 drugs (31%) had 731 incident supply chain issue reports. These were EDS-matched to 1597 unique drugs without concurrent exposures; 717 reports were matched to 10 comparators and 14 reports had 9 comparators, for a total sample size of 7296 comparators.

Per the Table, generic drugs were overrepresented in supply chain issue reports (616 reports [84%] vs 4397 comparison drugs [60%]), as were products at least 20 years old (375 reports [51%] vs 2434 comparison drugs [33%]), drugs with at least 5 manufacturers (378 reports [52%] vs 2225 comparison drugs [30%]), WHO essential medicines (316 reports [43%] vs 2199 comparison drugs [30%]), and drugs with less than $5 million baseline sales (234 reports [32%] vs 2158 comparison drugs [30%]). Manufacturers did not provide a reason for 264 reports (36%); the percentage of reports with unknown or unspecified reasons increased to 42% from February to April 2020 and 47% after May 2020 (eTable 3 in Supplement 1). Across the entire study period, more than half of reports were recalls or discontinuations (498 reports [68%]), mostly due to quality concerns (Table; eTable 4 in Supplement 1).

**Table.** Characteristics of Drugs With Supply Chain Issue Reports vs Matched Comparison Drugs, 2017-2021

Variable	Supply chain issue reports (n = 731)	Matched comparisons drugs (n = 7296)
Time period		
January 2017 to January 2020	489 (67)	4873 (67)
February to April 2020	57 (8)	570 (8)
May 2020 to September 2021	185 (25)	1847 (25)
Reporting agency^a^		
FDA	588 (80)	NA
ASHP	327 (45)	NA
Type of issue^a^		
Shortage probable	363 (50)	NA
Recall	131 (18)	NA
Discontinuation	367 (50)	NA
Supply chain issue duration, median (IQR), mo	3.7 (3.0 to 17.9)	NA
Duration category, mo		
<6	401 (55)	NA
6-11	85 (12)	NA
12-23	109 (15)	NA
24-35	57 (8)	NA
≥36	79 (11)	NA
Reason for supply chain issue^b^		
No or unspecified reason	264 (36)	NA
Manufacturing, packaging, or shipping issues	163 (22)	NA
Business decision	108 (15)	NA
Discontinuation of the manufacture of the drug	73 (10)	NA
Impurities or lack of sterility	55 (8)	NA
Other events	68 (9)	NA
Formulation		
Oral	403 (55)	4001 (55)
Parenteral	286 (39)	2652 (36)
Ophthalmic or otic	27 (4)	446 (6)
Inhaled	15 (2)	197 (3)
≥5 Manufacturers at baseline^c^	378 (52)	2225 (30)
WHO essential medicine	316 (43)	2199 (30)
Clinician-administered drug	197 (27)	1730 (24)
Marketing category		
Generic available	616 (84)	4397 (60)
Brand-name only	65 (9)	1812 (25)
Other or unknown	48 (7)	1046 (14)
Baseline sales <$5 million^c^	234 (32)	2158 (30)
Drug age, median (IQR), y	20.4 (13.8 to 30.7)	14.5 (7.8 to 23.5)
Time since market entry, y^d^		
<5	40 (5)	976 (13)
5-9	78 (11)	1468 (20)
10-19	238 (33)	2412 (33)
≥20	375 (51)	2434 (33)
Change in MIDAS units, relative to 3 mo before report issuance, mean (95% CI), %		
Quarter comprising 1-3 mo after report	−5.1 (−8.2 to −2.1)	NA
Quarter comprising 4-6 mo after report	−7.0 (−14.7 to 0.0)	NA

Abbreviations: ASHP, American Society of Health-Systems Pharmacists; FDA, US Food and Drug Administration; MIDAS, Multinational Integrated Data Analysis; NA, not applicable; WHO, World Health Organization.

^a^ Categories are not mutually exclusive since a single drug supply issue episode could include multiple events.

^b^ Other events included regulatory delays, shortages of ingredients, demand increases, or other reasons.

^c^ Measured in MIDAS for the 6 months immediately prior to supply issue event initiation (baseline period).

^d^ As documented in the August 2022 FDA National Drug Code Directory of currently available NDCs.

### Unadjusted Results

**Figure 2.**
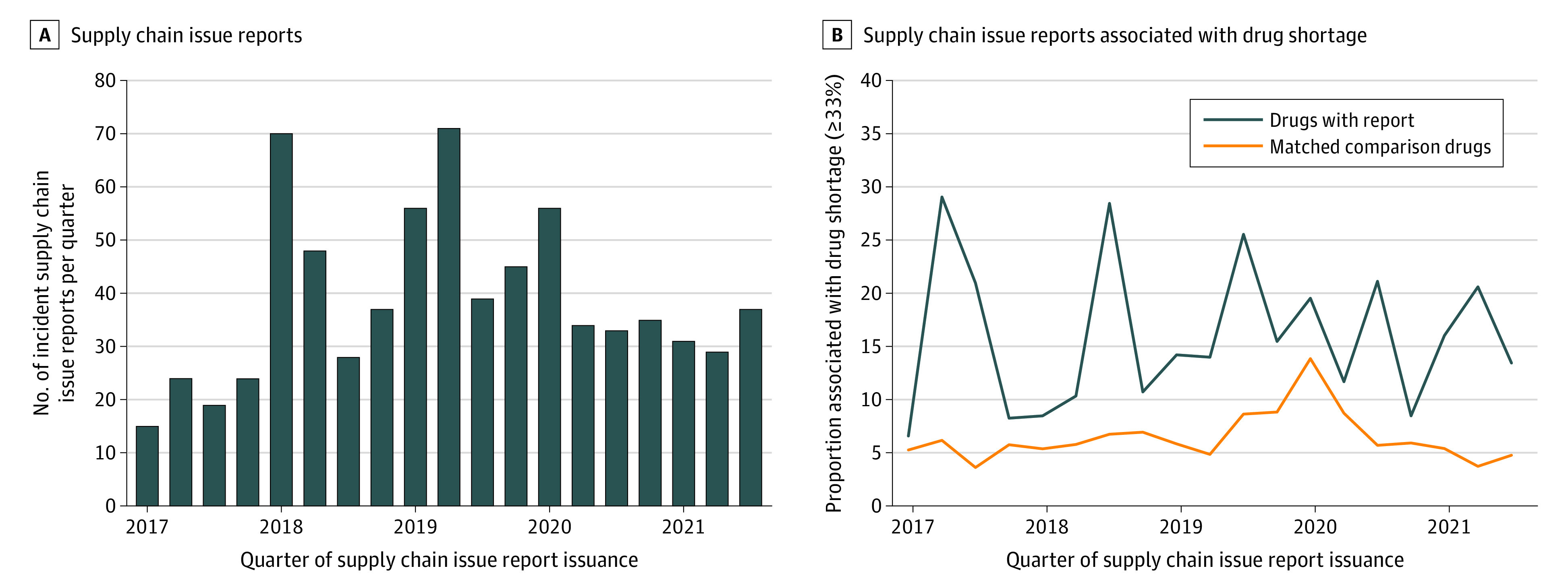
Unadjusted Proportion of Drugs With Incident Supply Chain Issue Reports and Matched Comparison Drugs With Meaningful (≥33%) Drug Shortages Within 6 Months, by Quarter, 2017-2021

We observed 15 to 71 incident supply chain issue reports per quarter from 2017 to 2021 (Figure 2). Across the study period, the mean change in drug purchases was −5.1% (95% CI, −8.2% to −2.1%) in the first quarter and −7.0% (95% CI, −14.7 to 0.01%) in the second quarter after report-issuance (Table). Among all reports, 113 reports (16%) were associated with shortages (≥33% decrease) within 6 months vs 491 of 7290 unexposed comparison drugs (7%) (eTable 5 in Supplement 1). We observed an increase in comparison drugs with shortages in the first quarter of 2020 (Figure 2). Severe shortages (≥66% decrease) were less common, with 63 reports (9%) and 188 comparison drugs (3%) associated with severe shortages (eTable 5, eFigure 4, and eFigure 5 in Supplement 1). Nonrecall and nondiscontinuation reports were more likely to be associated with meaningful (83 reports [23%]) and severe (43 reports [12%]) shortages, compared with recalls (meaningful: 13 reports [10%]; severe: 4 reports [3%]) and discontinuations (meaningful: 48 reports [13%]; severe: 33 reports [9%]) (eFigure 4 in Supplement 1).

### Adjusted Results

**Figure 3.**
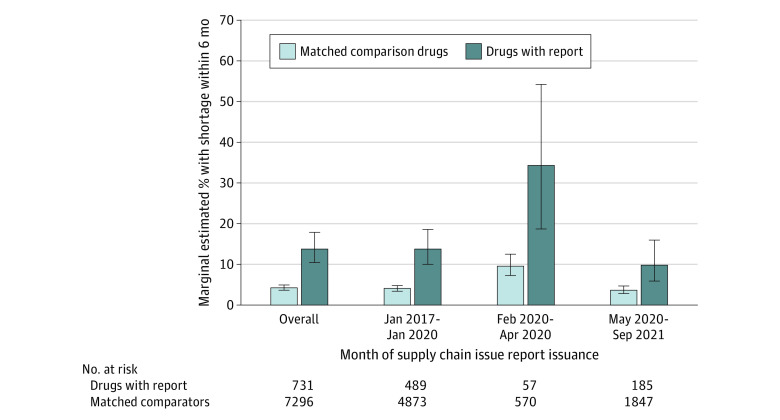
Adjusted Marginal Odds Ratios (mORs) and Estimated Probabilities of Meaningful (≥33%) Drug Shortages Within 6 Months Post hoc mORs and marginal estimated probabilities were obtained from the fitted logistic regression model. Full regression output is provided in eTable 5 in Supplement 1. Estimated probabilities represent absolute incidence outcomes. Estimated marginal outcomes were obtained by calculating the mean over drug formulation, years since US Food and Drug Administration approval, marketing category, World Health Organization essential medicine status, clinician vs self-administration, total sales, and number of manufacturers at baseline.

Marginal odds ratios (mORs) and estimated probabilities from our logistic regression analysis are shown in Figure 3, with full regression output in eTable 6 in Supplement 1. Across baseline drug characteristics, the marginal estimated percentage of supply chain issue reports associated with shortages was 13.7% (95% CI, 10.4%-17.8%) of reports vs 4.1% (95% CI, 3.6%-4.8%) of comparators (Figure 3). Compared with drugs without reports, drugs with reports had greater marginal odds of shortages (mOR, 3.7 [95% CI, 2.9-5.1]). Results were similar for severe shortages (mOR, 6.3 [95% CI, 3.7-10.9]), although adjusted incidences were lower (6.0% [95% CI, 3.8%-9.2%] for supply chain issue reports and 1.0% [95% CI, 0.7%-1.4%] for comparison drugs) (eFigure 6 in Supplement 1).

### Shortages Prior to vs During the COVID-19 Pandemic

As shown in Figure 3, the adjusted incidence of shortages for supply chain reports issued in the first 3 months of the COVID-19 pandemic were more likely to be associated with subsequent shortages than reports issued during the prepandemic period (34.2% [95% CI, 18.6%-54.1%] vs 13.7% [95% CI, 10.0%-18.5%]). However, we also observed increases in the adjusted proportion of comparison drugs without reports with shortages at or above our 33% cutoff compared with the prepandemic period (9.5% [95% CI, 7.2%-12.5%] vs 4.0% [95% CI, 3.3%-4.7%]). There were no significant interactions by COVID-19 pandemic period. The 95% CIs of the mORs comparing drugs with vs without reports issued from February to April 2020 overlapped with the prepandemic period (prepandemic mOR, 3.9 [95% CI, 2.6-5.6] vs February to April 2020 mOR, 4.9 [95% CI, 2.1-11.6]; *P* for interaction = .60). The mOR for drugs with vs without reports issued during or after May 2020 also did not significantly differ from the prepandemic period (mOR, 2.9 [95% CI, 1.6-5.3]; *P* for interaction = .38) (Figure 3). The association between report status and severe shortages did not significantly differ during the prepandemic period (mOR, 6.8 [95% CI, 3.6-12.4]) vs during the early stage of the pandemic (February to April 2020: mOR, 5.9 [95% CI, 1.3-26.3]) or after May 2020 (mOR, 5.3 [95% CI, 2.4-11.8]) (eFigure 6 in Supplement 1).

### Shortages by Characteristics

**Figure 4.**
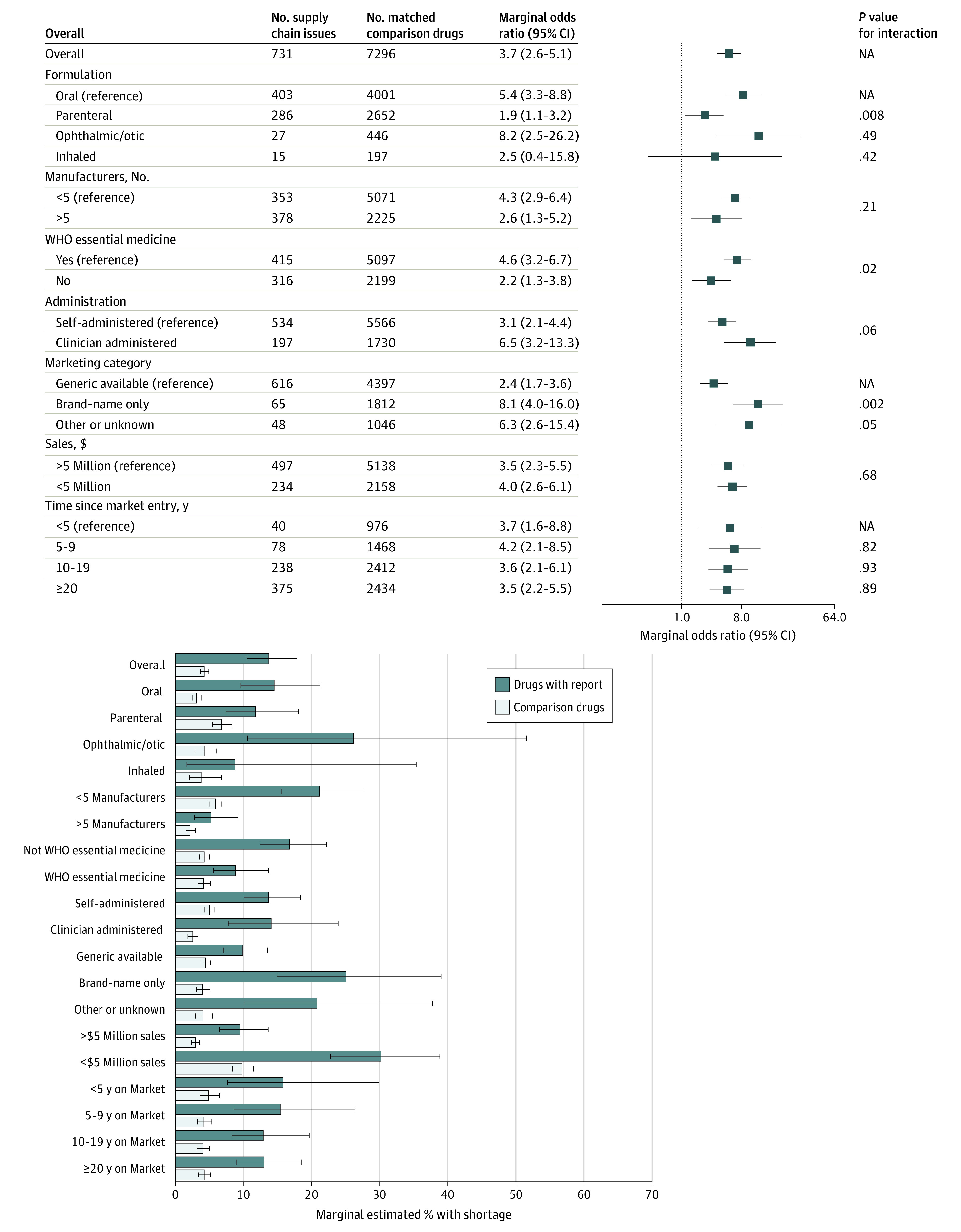
Adjusted Marginal Odds Ratios and Predicted Probabilities of Meaningful (≥33%) Drug Shortages Within 6 Months, Supply Chain Issue Reports vs Matched Comparison Drugs, by Drug Characteristic Post hoc mORs and marginal estimated probabilities were obtained from the fitted logistic regression model. Full regression output is provided in eTable 6 in Supplement 1. Estimated probabilities represent absolute incidence outcomes. Estimated marginal outcomes were obtained by calculating the mean over drug formulation, years since US FDA approval, marketing category, WHO essential medicine status, clinician vs self-administration, total sales, and number of manufacturers at baseline.

Figure 4 summarizes our assessment of heterogeneity by drug characteristics. Across other characteristics, we observed a significant interaction for parenteral drugs with vs without reports, compared with oral drugs (parenteral mOR, 1.9 [95% CI, 1.1-3.2] vs oral mOR, 5.4 [95% CI, 3.3-8.8]; *P* for interaction = .008). There were also significant interactions by WHO essential medicine status (essential mOR, 2.2 [95% CI, 1.3-5.2] vs nonessential mOR, 4.6 [95% CI, 3.2-6.7]; *P* for interaction = .02), and for brand-name vs generic drugs (brand-name mOR, 8.1 [95% CI, 4.0-16.0] vs generic mOR, 2.4 [95% CI, 1.7-3.6]; *P* for interaction = .002). We observed similar heterogeneity for severe shortages (eFigure 7 in Supplement 1).

### Sensitivity Analyses

As shown in eFigure 8 and eTables 7 to 11 in Supplement 1, sensitivity analyses were consistent with our main findings. Excluding recalls and discontinuations resulted in a slightly higher adjusted incidence of shortages (16.7% [95% CI, 12.9%-21.4%]); however, the marginal odds of shortage were similar (mOR, 3.9 [95% CI, 2.8-5.4]).

## Discussion

In this national cross-sectional study, approximately 1 in 7 supply chain issue reports (14%) were associated with drug shortages within 6 months, and 1 in 15 supply chain issue reports (6%) were associated with severe shortages. The incidence of shortages increased to 1 in 3 reports (34%) from February to April 2020 and then reduced to prepandemic levels after May 2020. However, we also observed increases in shortages in drugs with no supply chain issues during the early pandemic. WHO essential and parenteral medicines were less likely to be associated with shortages, and brand name drugs were more likely to be associated with shortages.

Our findings are mostly consistent with a similar RAND study in MIDAS from 2016 to 2019.^30^ In that study, utilization decreased by a mean of 8.4% in the quarter after a supply chain issue report vs 5.1% in our study. Our smaller absolute estimate may be due to a lower incidence of shortages after May 2020. Nevertheless, our study is an important contribution to the literature because we included recalls and discontinuations, which were less likely to be associated with shortages. We also tested whether shortages changed after the onset of the COVID-19 pandemic.

In our study, drugs with and without reports from February to April 2020 were more likely to be associated with subsequent shortages, compared with prepandemic rates. These findings are consistent with the known destabilizing impact of COVID-19 on supply chains. We observed a return to prepandemic levels after May 2020. This finding may be explained by the implementation of new policies that empowered the FDA to take more direct action.^16,40,41^ Despite these improvements, ongoing vulnerabilities in drug supply chains are in need of policy action,^17^ including greater supply chain transparency^19,21^ and addressing root economic causes of shortages.^42^

It is also possible that issuance of an FDA or ASHP report, while consistently associated with higher odds of shortages, may have underestimated relevant upstream issues at pandemic start. Before the pandemic, 4% of drugs with no report were associated with shortages at or above our 33% decrease cutoff; this increased to 10% from February to April 2020. We also observed a greater proportion of supply chain issues with no or unspecified reasons during the pandemic vs the prepandemic period. Temporary pauses in quality assurance reviews during COVID-19 lockdowns may have been associated with underreporting. There are no penalties for failure to report.^22^ Our wide 95% CIs for February to April 2020 should be interpreted with caution, given the small sample size (57 reports) and potential underreporting.

When examining variation across drugs, we observed a higher mOR for the association between having a report and subsequent shortage among branded medications. This finding is not unexpected, given that branded markets usually have 1 supplier. The higher mOR among branded medications may therefore be explained by the unavailability of alternative manufacturers to supply product. Shortages of branded drugs may also respond to different underlying reasons, such as increased demand, quotas, or manufacturer capacity.^43,44^ In contrast, approximately 4 in 5 supply chain issue reports in our study were for generic medications, and one-third were for drugs with prereport sales less than $5 million. Generic supply chain issues likely involve insufficient incentives for manufacturers to invest in resilient supply chains for products of limited profitability.^11,41^ In this context, recent policy proposals suggest the implementation of reimbursement models which would provide add-on payments for generics manufactured in more resilient supply chains.^42,45^ Our findings of differing shortage risk by supply chain characteristics could be used to develop lists of high-priority generic drugs for the application of these novel reimbursement models. Our findings may also be informative for the development of shortage-critical lists to prioritize medications for other drug shortage policies,^22,46^ like those in Canada and Europe.^47,48^ Similar frameworks have not yet been adopted in the US but would be useful in the prioritization of national drug shortage policy.^49^

Interestingly, we observed a lower mOR for the association between having a report and subsequent shortage for parenteral vs oral drugs. Changes in demand for these products, eg, cancellation of elective surgeries, may have resulted in residual supply during the pandemic. A higher proportion of parenteral drugs without supply chain reports were also associated with meaningful decreases in supply at or above our 33% cutoff, eg, 7% of parenteral vs 3% of oral drugs, suggesting that it is also possible all parenteral products were more vulnerable to disruption, lessening the difference between exposed vs unexposed drugs.^11,41^ Generic sterile injectables have therefore been prioritized in several recent drug shortage policy proposals.^42,45^

### Limitations

There are several limitations to this study. First, the observed increase in drugs without supply chain issue reports that experienced shortages from February to April 2020 suggests potential for underreporting during the COVID-19 shutdown. Our mORs comparing drugs with vs without reports from February to April 2020 should be interpreted with caution. Our dataset was also time-limited (2017-2021). Many pandemic-related policies were not yet fully implemented, and we could not assess the association of the end of the public health emergency in May 2023.^50^ Second, although MIDAS is the most comprehensive dataset available, it does not fully capture over-the-counter and topical products, many of which have recently been experiencing shortages. Third, we do not know the downstream impact of the identified shortages on patients. Bedside efforts and availability of therapeutic alternatives may maintain standards of care, even when shortages occur.^22^ ASHP includes information on equivalents for some drugs; however, this information was lacking in FDA databases.

## Conclusions

This cross-sectional study found that 1 in 7 supply chain issue reports were associated with drug shortages. Although the shortages increased early in the COVID-19 pandemic, we observed a return to prepandemic levels after May 2020. As the public health emergency ends, continued policy effort is needed to mitigate shortages and to prepare pharmaceutical supply chains for future, unanticipated shocks.

## Notes

Supplement 1. **eFigure 1.** Conceptual Model **eTable 1.** Excluded Nonprescription Products **eFigure 2.** Merging Algorithm for FDA and ASHP Supply Issue Reports **eFigure 3.** Examples of Medications With No Supply Decrease, Meaningful (≥33%) Shortage and Severe (≥66%) Shortage in MIDAS **eTable 2.** Covariate Definitions **eTable 3.** Characteristics of Drugs with Supply Chain Issue Reports, by COVID-19 Pandemic Period **eTable 4.** Characteristics of Drugs with Supply Chain Issue Reports, by Report Type **eTable 5. **Characteristics of Supply Chain Issue Reports and Matched Comparison Drugs, by Shortage Status **eFigure 4. **Unadjusted Probabilities of Meaningful (≥33%) and Severe (≥66%) Drug Shortages Within 6 Months, by Report Type **eFigure 5.** Unadjusted Proportion of Incident Supply Chain Issue Reports and Matched Comparison Drugs Associated With Severe (≥66%) Drug Shortages Within 6 Months, by Quarter, 2017-2021 **eFigure 6.** Marginal Odds Ratios and Estimated Probabilities of Severe (≥66%) Shortages Within 6 Months, Supply Chain Issue Reports vs Matched Comparison Drugs, Prepandemic vs During the COVID-19 Pandemic **eFigure 7.** Marginal Odds Ratios and Estimated Probabilities of Severe (≥66%) Shortages Within 6 Months, Supply Chain Issues vs Matched Comparison Drugs, by Drug Characteristics **eTable 6.** Odds of Meaningful (≥33%) and Severe (≥66%) Shortages for Supply Chain Issue Reports vs Matched Comparison Drugs **eFigure 8.** Marginal Odds Ratios and Estimated Probabilities of Meaningful (≥33%) and Severe (≥66%) Shortages for Supply Chain Issue Reports vs Matched Comparison Drugs, Sensitivity Analyses Using Different Outcome Definitions and Inclusion Criteria **eTable 7.** Odds of Meaningful (≥33%) and Severe (≥66%) Shortages for Supply Chain Issue Reports vs Matched Comparison Drugs, Sensitivity Analysis Using 9 Months to Define Shortages **eTable 8.** Odds of Meaningful (≥33%) Shortages for Supply Chain Issue Reports vs Matched Comparison Drugs, Sensitivity Analyses Using Different Inclusion Criteria **eTable 9.** Odds of Severe (≥66%) Shortages for Supply Chain Issue Reports vs Matched Comparison Drugs, Sensitivity Analyses Using Different Inclusion Criteria **eTable 10.** Characteristics of Supply Chain Issue Reports vs Matched Comparison Drugs, 2017-2021, Sensitivity Analysis Among Propensity-Score-Matched Cohort **eTable 11.** Odds of Meaningful (≥33%) and Severe (≥66%) Shortages for Supply Chain Issue Reports vs Matched Comparison Drugs, Sensitivity Analysis Among Propensity-Matched Cohort

Supplement 2.
Data Sharing Statement
