# Supplementary material for: Drug Shortages Prior to and During the COVID-19 Pandemic
Source: JAMA Netw Open. 2024 Apr 5;7(4):e244246. doi: 10.1001/jamanetworkopen.2024.4246 (PMC10998160; doi:10.1001/jamanetworkopen.2024.4246)
Supplement: Supplement 1. — eFigure 1. Conceptual Model eTable 1. Excluded Nonprescription Products eFigure 2. Merging Algorithm for FDA and ASHP Supply Issue Reports eFigure 3. Examples of Medications With No Supply Decrease, Meaningful (≥33%) Shortage and Severe (≥66%) Shortage in MIDAS eTable 2. Covariate Definitions eTable 3. Characteristics of Drugs with Supply Chain Issue Reports, by COVID-19 Pandemic Period eTable 4. Characteristics of Drugs with Supply Chain Issue Reports, by Report Type eTable 5. Characteristics of Supply Chain Issue Reports and Matched Comparison Drugs, by Shortage Status eFigure 4. Unadjusted Probabilities of Meaningful (≥33%) and Severe (≥66%) Drug Shortages Within 6 Months, by Report Type eFigure 5. Unadjusted Proportion of Incident Supply Chain Issue Reports and Matched Comparison Drugs Associated With Severe (≥66%) Drug Shortages Within 6 Months, by Quarter, 2017-2021 eFigure 6. Marginal Odds Ratios and Estimated Probabilities of Severe (≥66%) Shortages Within 6 Months, Supply Chain Issue Reports vs Matched Comparison Drugs, Prepandemic vs During the COVID-19 Pandemic eFigure 7. Marginal Odds Ratios and Estimated Probabilities of Severe (≥66%) Shortages Within 6 Months, Supply Chain Issues vs Matched Comparison Drugs, by Drug Characteristics eTable 6. Odds of Meaningful (≥33%) and Severe (≥66%) Shortages for Supply Chain Issue Reports vs Matched Comparison Drugs eFigure 8. Marginal Odds Ratios and Estimated Probabilities of Meaningful (≥33%) and Severe (≥66%) Shortages for Supply Chain Issue Reports vs Matched Comparison Drugs, Sensitivity Analyses Using Different Outcome Definitions and Inclusion Criteria eTable 7. Odds of Meaningful (≥33%) and Severe (≥66%) Shortages for Supply Chain Issue Reports vs Matched Comparison Drugs, Sensitivity Analysis Using 9 Months to Define Shortages eTable 8. Odds of Meaningful (≥33%) Shortages for Supply Chain Issue Reports vs Matched Comparison Drugs, Sensitivity Analyses Using Different Inclusion Criteria eTable 9. Odds of Se [file jamanetwopen-e244246-s001.pdf]

Supplemental Online Content

Callaway Kim K, Rothenberger SD, Tadrous M, et al. Drug shortages prior to and during the COVID-19 pandemic. *JAMA Netw Open*. 2024;7(4):e244246. doi:10.1001/jamanetworkopen.2024.4246

eFigure 1. Conceptual Model
eTable 1. Excluded Nonprescription Products
eFigure 2. Merging Algorithm for FDA and ASHP Supply Issue Reports
eFigure 3. Examples of Medications With No Supply Decrease, Meaningful (≥33%) Shortage and Severe (≥66%) Shortage in MIDAS
eTable 2. Covariate Definitions
eTable 3. Characteristics of Drugs with Supply Chain Issue Reports, by COVID-19 Pandemic Period
eTable 4. Characteristics of Drugs with Supply Chain Issue Reports, by Report Type
eTable 5. Characteristics of Supply Chain Issue Reports and Matched Comparison Drugs, by Shortage Status
eFigure 4. Unadjusted Probabilities of Meaningful (≥33%) and Severe (≥66%) Drug Shortages Within 6 Months, by Report Type
eFigure 5. Unadjusted Proportion of Incident Supply Chain Issue Reports and Matched Comparison Drugs Associated With Severe (≥66%) Drug Shortages Within 6 Months, by Quarter, 2017-2021
eFigure 6. Marginal Odds Ratios and Estimated Probabilities of Severe (≥66%) Shortages Within 6 Months, Supply Chain Issue Reports vs Matched Comparison Drugs, Prepandemic vs During the COVID-19 Pandemic
eFigure 7. Marginal Odds Ratios and Estimated Probabilities of Severe (≥66%) Shortages Within 6 Months, Supply Chain Issues vs Matched Comparison Drugs, by Drug Characteristics
eTable 6. Odds of Meaningful (≥33%) and Severe (≥66%) Shortages for Supply Chain Issue Reports vs Matched Comparison Drugs
eFigure 8. Marginal Odds Ratios and Estimated Probabilities of Meaningful (≥33%) and Severe (≥66%) Shortages for Supply Chain Issue Reports vs Matched Comparison Drugs, Sensitivity Analyses Using Different Outcome Definitions and Inclusion Criteria
eTable 7. Odds of Meaningful (≥33%) and Severe (≥66%) Shortages for Supply Chain Issue Reports vs Matched Comparison Drugs, Sensitivity Analysis Using 9 Months to Define Shortages
eTable 8. Odds of Meaningful (≥33%) Shortages for Supply Chain Issue Reports vs Matched Comparison Drugs, Sensitivity Analyses Using Different Inclusion Criteria
eTable 9. Odds of Severe (≥66%) Shortages for Supply Chain Issue Reports vs Matched Comparison Drugs, Sensitivity Analyses Using Different Inclusion Criteria
eTable 10. Characteristics of Supply Chain Issue Reports vs Matched Comparison Drugs, 2017-2021, Sensitivity Analysis Among Propensity-Score-Matched Cohort
eTable 11. Odds of Meaningful (≥33%) and Severe (≥66%) Shortages for Supply Chain Issue Reports vs Matched Comparison Drugs, Sensitivity Analysis Among Propensity-Matched Cohort

This supplemental material has been provided by the authors to give readers additional information about their work.

© 2024 Callaway Kim K et al. *JAMA Network Open.*

e-Figure 1: Conceptual Model[a]

e-Figure 1 presents the conceptual model for our study, which was modified from "Building Resilience into the Nation's Medical Product Supply Chains" (2022), Committee on Security of America's Medical Product Supply Chain, Board on Health Sciences, Policy Health and Medicine Division, National Academies of Science, Engineering, and Medicine. Since 2012, companies must report to the FDA known issues affecting the supply chain which could result in shortages (hereafter, supply chain issue reports). These reports are also tracked by the American Society of Health-Systems Pharmacists (ASHP). However, supply chain issue reports may be incomplete indicators for meaningful decreases in national supplies (i.e., drug shortages). As represented by the blue pentagon in Scenario A, protective "shields" in drug supply chains – including opening more production lines, importing similar drugs from other countries, lengthening expiration dates or using residual supplies - may address issues prior to the depletion of supplies held by manufacturers, wholesalers, and pharmacies. Not all reports therefore necessarily result in drug shortages. The white circle in Scenario B represents supply chain failures (i.e., "holes" in the "protective shield") through which upstream reports may be associated with subsequent drug shortages. This is our primary outcome measure for this study and is consistent with FDA and ASHP's definition of drug shortages as meaningful decreases in supply with potential to impact patient care.

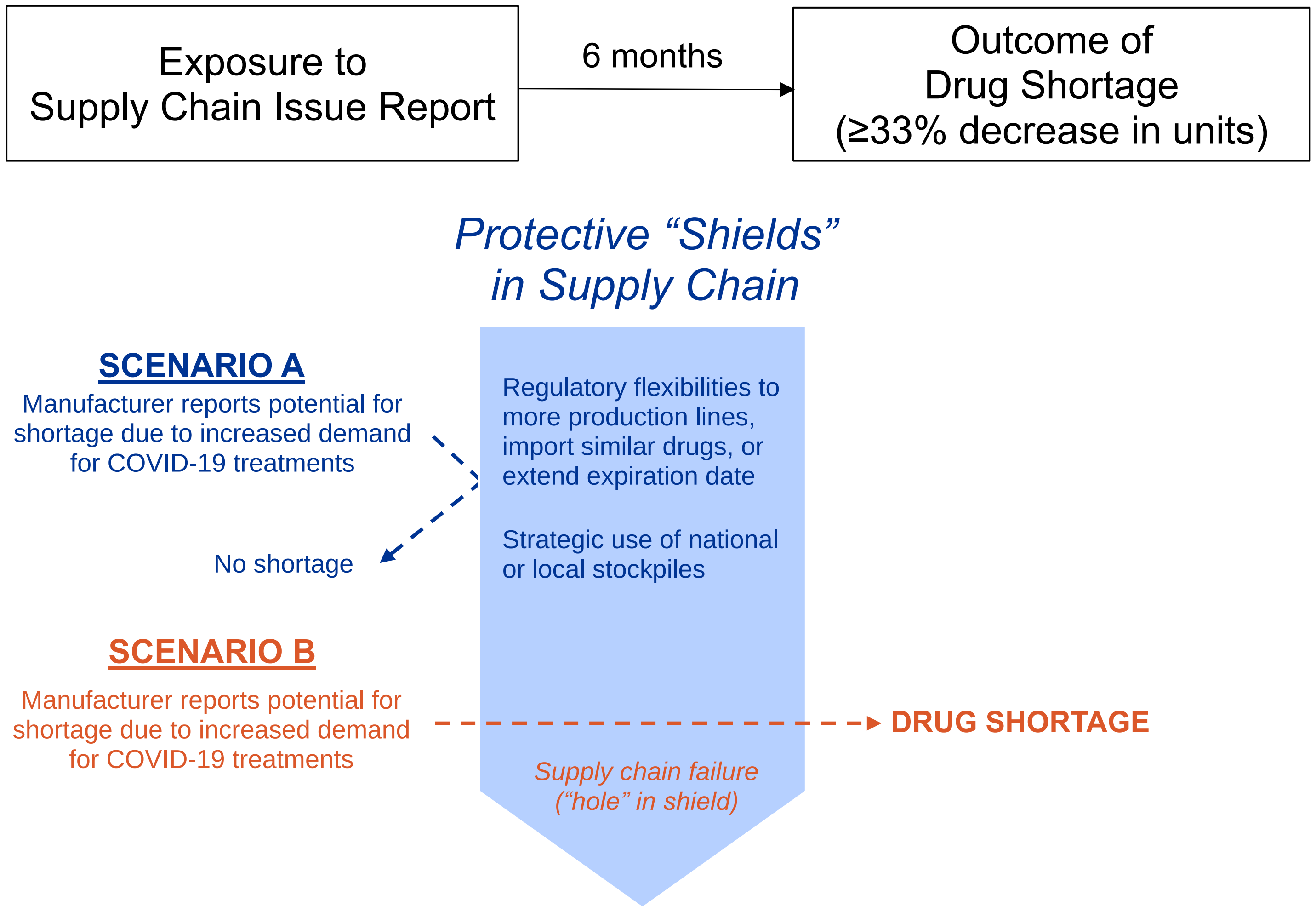

© 2024 Callaway Kim K et al. *JAMA Network Open.*

e-Table 1: Excluded Non-Prescription Products

e-Table 1 presents a full list of non-prescription products in MIDAS which were excluded from our study. MIDAS is the most comprehensive pharmacy purchasing dataset available in the United States, and includes 85% of retail and 97% of non-retail purchases by U.S. pharmacies from wholesalers and manufacturers, based on unit volume. However, it may not include all purchases made outside of pharmacy settings, including those for over-the-counter products or products with unique supply chains (e.g., radiopharmaceuticals, diagnostic tests). We therefore excluded all over-the-counter products from our study. We also excluded topicals (e.g., dermatologic formulations), many of which are over-the-counter. Topical dosages are also difficult to compare to other systemic formulations, meaning that the unit volumes in MIDAS may not be comparable to other included drugs.

Reason for Exclusion	Exclusion Rule
Products with unknown, non-human, or topical formulations	• *Oral topical products*: New Form Codes starting with “K” or “ACS”, “ASB”, “AYG”, “DEH”, “DEK”<br>• *External topical, dermatological, or hemorrhoid products*: New Form Codes starting with “M”<br>• *Rectal products*: New Form Codes starting with “H”<br>• *Nasal topical products*: New Form Codes “IEP”, “IGB”, “IGN”, “IGP”, “IVP” or ATC3 “R1A TOPICAL NASAL PREPS”<br>• *Ointments*: New Form Codes “JGP”, “JSA”, “JSN”, “QSA”, “QYM”<br>• *Transdermal products*: New Form Codes “JWM”, “JWN”<br>• *Vaginal products*: New Form Codes starting with “T”<br>• *Products with unknown formulation:* New Form Codes starting with “Z”<br>• *Non-human products*: New Form Codes starting with “V”
Over-the-counter products	• *Over-the-counter oral antacids and anti-flatulent products*: New Form Codes starting with “A”, “B”, “D”, or “EEK”, “EEP”, “EGA”, “EGM”, “JGA”, “JGE”, “JRP”, “JVA”, “JVN”, “JVP” AND ATC3 code in “A2A ANTACIDS ANTIFLATULENTS”, “A2X OTHER STOMACH DISORDER PRODUCTS” EXCEPT products available with a prescription (molecule list “NIZATIDINE”)<br>• *Over-the-counter drugs for constipation or diarrhea*: New Form Codes starting with “A”, “B”, “D”, or “EEK”, “EEP”, “EGA”, “EGM”, “JGA”, “JGE”, “JRP”, “JVA”, “JVN”, “JVP” AND ATC3 code in “A6A DRUGS FOR CONSTIPATION”, “A7H MOTILITY INHIBITORS”, “A7B INTEST ADSORBENT ANTIDIA”, “A7F ANTIDIARR MICRO-ORGANISM” EXCEPT products available with a prescription (molecule list in “ALVIMOPAN”, “LINACLOTIDE”, “LUBIPROSTONE”, “MACROGOL(S)”, “METHYLNALTREXONE BROMIDE”, “NALDEMEDINE”, “NALOXEGOL”, “PRUCALOPRIDE”)<br>• *Over-the-counter oral analgesics*: New Form Codes starting with “A”, “B”, “D”, or “EEK”, “EEP”, “EGA”, “EGM”, “JGA”, “JGE”, “JRP”, “JVA”, “JVN”, “JVP” AND [ATC3 code “N2B NON-NARCOTIC ANALGESICS” OR molecule list in “PARACETAMOL”, “ACETYLSALICYLIC ACID!CAFFEINE!PARACETAMOL”, “ACETYLSALICYLIC ACID!CAFFEINE!SALICYLAMIDE”, “ACETYLSALICYLIC ACID”, “ACETYLSALICYLIC ACID!CALCIUM”, “IBUPROFEN”, “CAFFEINE!PARACETAMOL”, “METHENAMINE!SALICYLIC ACID”] EXCEPT products available with a prescription (molecule list in “ACETYLSALICYLIC ACID!BUTALBITAL!CAFFEINE”, “BUTALBITAL!CAFFEINE!PARACETAMOL”, “BUTALBITAL!PARACETAMOL”, “CAFFEINE!ISOMETHEPTENE!PARACETAMOL”, “DICLOFENAC”, “DIFLUNISAL”, “DIPHENHYDRAMINE!IBUPROFEN”, “DIPHENHYDRAMINE!NAPROXEN”, “INDOMETACIN”, “KETOROLAC”, “NALOXONE!PENTAZOCINE”, “NAPROXEN”, “PARACETAMOL!TRAMADOL”, “TRAMADOL”)

© 2024 Callaway Kim K et al. *JAMA Network Open.*

	• *Menstrual pain or other over-the-counter gynecologic products:* New Form Codes starting with “A”, “B”, “D”, or “EEK”, “EEP”, “EGA”, “EGM”, “JGA”, “JGE”, “JRP”, “JVA”, “JVN”, “JVP” AND molecule list in “PAMABROM”, “PAMABROM!PARACETAMOL”, “CAFFEINE!MEPYRAMINE”, “CAFFEINE!MEPYRAMINE!PARACETAMOL”, “MEPYRAMINE!PAMABROM!PARACETAMOL”, “ACETYLSALICYLIC ACID!CAFFEINE!CINNAMEDRINE”, “FOLIC ACID!INOSITOL”, “INOSITOL”, “INOSITOL NICOTINATE”<br>• *Over-the-counter nasal decongestants, cold or allergy preparations, expectorants, or antitussives*: [ATC3 in “R1B SYSTEMIC NASAL PREPS”, “R5A COLD PREPARATIONS”, “R5C EXPECTORANTS”, “R5D ANTITUSSIVES”, “R6A ANTIHISTAMINES SYSTEMIC”, “R5C EXPECTORANTS”, “R4A CHEST RUBS & INHALANTS”, “R7A RESPIRATORY STIMULANTS”, “R7X ALL OTH RESP SYST PRODS” OR molecule list in “DIPHENHYDRAMINE”, “RACEPINEFRINE”, “GUAIFENESIN!THEOPHYLLINE”,] EXCEPT products available with a prescription (molecule list in “ACETYLCYSTEINE”, “ACRIVASTINE!PSEUDOEPHEDRINE”, “BENZONATATE”, “CETIRIZINE!PSEUDOEPHEDRINE”, “CHLORPHENAMINE!GUAIFENESIN!HYDROCODONE!PSEUDOEPHEDRINE”, “CHLORPHENAMINE!HYDROCODONE”, “CHLORPHENAMINE!HYDROCODONE!PSEUDOEPHEDRINE”, “CHLORPHENAMINE!IBUPROFEN!PSEUDOEPHEDRINE”, “CODEINE!PHENYLEPHRINE!PROMETHAZINE”, “CODEINE!PROMETHAZINE”, “DESLORATADINE!PSEUDOEPHEDRINE”, “DORNASE ALFA”, “DOXAPRAM”, “FEXOFENADINE!PSEUDOEPHEDRINE”, “GUAIFENESIN!HYDROCODONE”, “HOMATROPINE!HYDROCODONE”, “IBUPROFEN!PHENYLEPHRINE”, “IBUPROFEN!PSEUDOEPHEDRINE”, “LORATADINE!PSEUDOEPHEDRINE”, “NAPROXEN!PSEUDOEPHEDRINE”, “PHENYLEPHRINE!PROMETHAZINE”)<br>• *Over-the-counter anti-emetics and anti-nausea drugs*: ATC3 in ”A4A ANTIEMETCS+ANTINAUSEANTS" EXCEPT products available with a prescription (molecule list not in "APREPITANT", "DIMENHYDRINATE", "DOXYLAMINE!PYRIDOXINE", "DRONABINOL", "FOSAPREPITANT", "FOSNETUPITANT!PALONOSETRON", "GRANISETRON", "ONDANSETRON", "PALONOSETRON", "PROCHLORPERAZINE", "ROLAPITANT", "TRIMETHOBENZAMIDE")<br>• *Over-the-counter eye and ear products*: ATC3 in “S1L CONTACT LENS SOLUTIONS”, “S1K DRY EYE PRODUCTS”, “S1G OCUL.A-ALLER.DECON.A-SEP”, “S1H LOCAL ANAESTHETICS - EYE”, “S1F MYDRIATICS+CYCLOPLEGICS “, “S1X OTHER OPHTHALMOLOGICALS”, "S2D OTHER EAR PREPARATIONS" EXCEPT products available with a prescription (molecule list “ALCAFTADINE”, “AZELASTINE”, “BEPOTASTINE”, “BRIMONIDINE”, “CICLOSPORIN”, “CROMOGLICIC ACID”, “CYCLOPENTOLATE”, “EMEDASTINE”, “EPINASTINE”, “HYDROXYAMPHETAMINE!TROPICAMIDE”, “HYPROLOSE”, “KETOROLAC!PHENYLEPHRINE”, “KETOTIFEN”, “LIDOCAINE”, “LODOXAMIDE”, “MACROGOL(S)!NAPHAZOLINE”, “NEDOCROMIL”, “OLOPATADINE”, “PHENYLEPHRINE”, “POVIDONE-IODINE”, “PROXYMETACAINE”, “TROPICAMIDE”, “ACETIC ACID”)
Oral vitamins, supplements, and nutritional products	• *Oral multivitamins and minerals*: New Form Codes starting with “A”, “B”, “D”, or “EEK”, “EEP”, “EGA”, “EGM”, “JGA”, “JGE”, “JRP”, “JVA”, “JVN”, “JVP” AND [ATC3 codes in “A11A MULTIVITAMINS + MINERALS”, “A11B MULTIVIT WITHOUT MINERAL”, “A11C VIT A & D INC. COMBS”, “A11E VITAMIN B COMPLEX”, “A11F VITAMIN B12 PLAIN”, “A11G VIT C INC.MINERAL COMBS”, “A11X OTHER VITAMINS”, “A12C OTHR MINERAL SUPPLEMENTS”, “B3A HAEMATINICS.IRON & COMBS”, “B3X OTH ANTI-ANAE+FOLIC ACID”, “S1M EYE TONICS&EYE VITAMINS” OR molecule list in “MULTIVITAMINS AND MINERALS”, "CALCIUM", "ZINC", “IRON FERRIC”, “NICOTINAMIDE”, “CALCIUM!FOLIC ACID!MAGNESIUM”)]<br>• *Oral nutritional supplements, probiotics, medical food, and natural products*: New Form Codes starting with “A”, “B”, “D”, or “EEK”, “EEP”, “EGA”, “EGM”, “JGA”, “JGE”, “JRP”, “JVA”, “JVN”, “JVP” AND [ATC3 codes in “A8A A-OBESITY PREPS EXC DIET”, “A12A CALCIUM”, “A12B POTASSIUM”, “V6A SLIMMING PREPARATIONS”, “V6B PROTEIN SUPPLEMENTS”, “V6D OTHER NUTRIENTS”, “V6C INFANT FORMULAS” OR molecule list in “GLUCOSE”, "FISH", "PROBIOTICS", "SPECIAL DIET PREPARATIONS", "GENERAL NUTRIENTS", "SUCROSE", "DOCONEXENT," "DOCONEXENT!ETHYL-EICOSAPENT", "DOCONEXENT!ICOSAPENT!PHYTOSTEROLS (UNSPECIFIED) ", "DOCONEXENT!ICOSAPENT!PHOSPHATIDYL SERINE", "DOCONEXENT!ICOSAPENT", "UBIQUINOL", "DIOSMIN", "GLUCOSAMINE", “VACCINIUM MACROCARPON”, “BIFIDOBACTERIUM BREVE!BIFIDOBACTERIUM INFANTIS!BIFIDOBACTERIUM LONGUM!LACTOBACILLUS ACIDOPHILUS!LACTOBACILLUS BULGARIS!LACTOBACILLUS

© 2024 Callaway Kim K et al. *JAMA Network Open.*

	PARACASEI!LACTOBACILLUS PLANTARUM!STREPTOCOCCUS THERMOPHILUS", "ASCORBIC ACID!ECHINACEA PURPUREA!FORSYTHIA SUSPENSA!GLUTAMINE!ISATIS TINCTORIA!LONICERA!LYSINE!MAGNESIUM!MANGANESE!RETINOL!SCHIZONEPETA!SELENIOUS ACID!VITAMIN E!VITEX AGNUS-CASTUS!ZINC!ZINGIBER OFFICINALE", "STARCH!SUCRALOSE", "GELATIN", "XANTHAN GUM", "CHLOROPHYLL", "FLUORINE", "COLECALCIFEROL!GENISTEIN!ZINC", "CALCIUM!COLECALCIFEROL!GENISTEIN!HYDROXYAPATITE!MENAQUINONE-7!ZINC", "ASCORBIC ACID!CHONDROITINSULFURIC ACID!GLUCOSAMINE", "B>-SITOSTEROL!BRASSICA CAMPESTRIS!CALCIUM!CAMPESTEROL!COLECALCIFEROL!CUCURBITA PEPO!CYANOCOBALAMIN!FOLIC ACID!LYCOPENE!MAGNESIUM!PYGEUM AFRICANUM!SELENIUM!SERENOA REPENS!STIGMASTEROL!URTICA DIOICA!VITAMIN E!ZINC", "ASCORBIC ACID!BETACAROTENE!BIOTIN!COLECALCIFEROL!FOLIC ACID!NICOTINIC ACID!PANTOTHENIC ACID!PYRIDOXINE!RETINOL!RIBOFLAVIN!THIAMINE!VACCINIUM MACROCARPON!VITAMIN E", "ASCORBIC ACID!BACILLUS COAGULANS!PHOSPHORIC ACID!VACCINIUM MACROCARPON", "ASCORBIC ACID!BACILLUS COAGULANS!PHENAZOPYRIDINE!VACCINIUM MACROCARPON", "ACETYLCYSTEINE!LEVOMEFOLIC ACID!MECOBALAMIN!SCHIZOCHYTRIUM", "CUCURBITA PEPO!CYANOCOBALAMIN!FOLIC ACID!LYCOPENE!MAGNESIUM!PYGEUM AFRICANUM!PYRIDOXINE!SELENIUM!SERENOA REPENS!VITAMIN E!ZINC", "COPPER!METHYLFOLATE!NICOTINAMIDE!ZINC", "ASCORBIC ACID!COPPER!FOLIC ACID!MAGNESIUM!NICOTINAMIDE!PYRIDOXINE!THIOCTIC ACID!ZINC", "AZELAIC ACID!COPPER!FOLIC ACID!NICOTINAMIDE!PYRIDOXINE!ZINC", "UBIQUINONE(S)")]
Products with unique supply chains	• *Radio pharmaceuticals and diagnostic imaging agents*: ATC3 in "S1T OPHTHALMOL DIAG AGENTS", "T1B IONIC ANGIO-UROGRAPHY", "T1C GASTROENTEROGRAPHY", "T1E MRI AGENTS", "T1X OTHER IMAGING AGENTS", "V3C RADIO PHARMACEUTICALS"<br>• *Antidotes and allergens*: ATC3 in "V3E ANTIDOTES" OR molecule list in "CHARCOAL!SORBITOL", "CHARCOAL"<br>• *Allergens*: ATC3 in "V1A ALLERGENS"<br>• *Diagnostic tests*: ATC3 in "T2C PREGNANCY&OVULATION TEST", "T2D DIABETES TESTS", "T2X ALL OTH DIAGNOSTIC TEST"
Other excluded products	• *Non-specific products:* molecule list in "COMPOSITION UNKNOWN", "DIURETICS AND URINARY PREPARATION"<br>• *Unapproved products or products not used in the United States:* molecule list in "HYOSCYAMINE", "HYOSCYAMINE!METHENAMINE!METHYLTHIONINIUM!PHOSPHORIC ACID", "HYOSCYAMINE!METHENAMINE!METHYLTHIONINIUM!PHOSPHORIC ACID!SALICYLIC ACID", "HYOSCYAMINE!METHENAMINE!PHOSPHORIC ACID!SALICYLIC ACID", "ATROPINE!HYOSCYAMINE!PHENOBARBITAL!SCOPOLAMINE", "QUININE", "PYRANTEL", "PHYSOSTIGMINE", "MELATONIN", "HOMOEOPATHIC MEDICINES", "FUMARIC ACID!NICKEL!POTASSIUM"<br>• *Other miscellaneous excluded drug classes:* ATC3 in "V3D DETOX AG A-NEOPLAST TRMT", "A6B BOWEL CLEANSERS", "N7B ANTISMOKING PRODUCTS", "A7G ORAL ELECTROL. REPLACER", "S1S OPHTHALMOL SURGICAL AIDS"

© 2024 Callaway Kim K et al. *JAMA Network Open.*

e-Figure 2: Merging Algorithm for FDA and ASHP Supply Issue Reports

e-Figure 2 provides three hypothetical examples of our merging algorithm to define unique supply chain issue report episodes from the multiple public databases used in this study. Our inclusion of supply chain issue reports from FDA's Drug Shortage website (herein, "FDA-probable shortages"), ASHP's website (herein, "ASHP-reported probable shortage) and FDA-reported recalls and discontinuations is an innovation of our study.

As shown in Example #1, we considered reports for the same drug-form from different websites to be within the same reporting episode if they had ≥1 day of overlap. As shown in Example #2, we also merged reports which did not overlap but which were <90 days from each other. This allowed for differences in FDA and ASHP reporting, including slightly different definitions of drug shortages.

As shown in Example #3, we assigned recalls and discontinuations a length equal to 90 days. Recalls and discontinuations are by-definition indefinite so often have a missing end date. However, based on our clinical experience and communications with suppliers and providers, we anticipated that the largest impact on supply for recalls and discontinuations was likely to occur within 3 months (90 days). This period is close enough in time t for supply decreases (i.e., shortages) to be attributed to the precipitating recall or discontinuation event, while also allowing for potential exhaustion of typical back-stock levels.

Later in this Supplement, we present sensitivity analyses which used the FDA and ASHP dataset separately, as well as excluded recalls and discontinuations from our analysis.

<u>Example #1:</u> Shortages Overlapping by ≥1 day

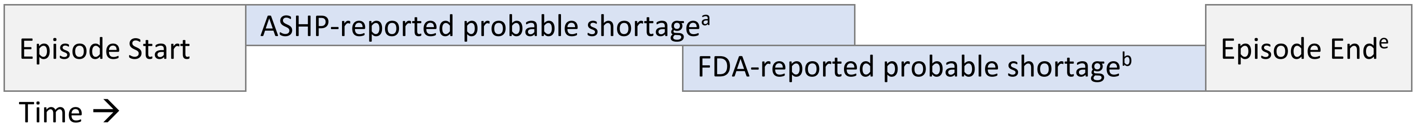

<u>Example #2</u>: Shortages <90 days Apart

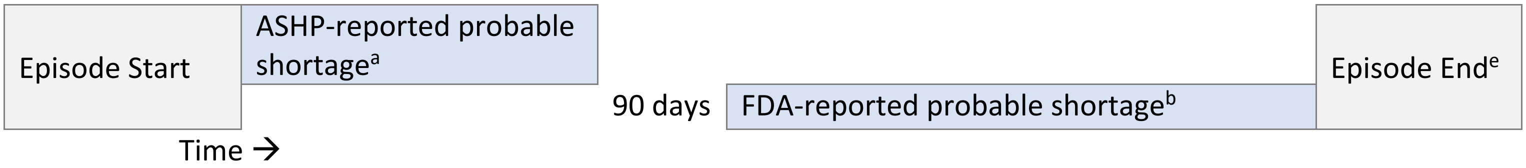

<u>Example #3:</u> FDA-reported Discontinuation and Recall Reports

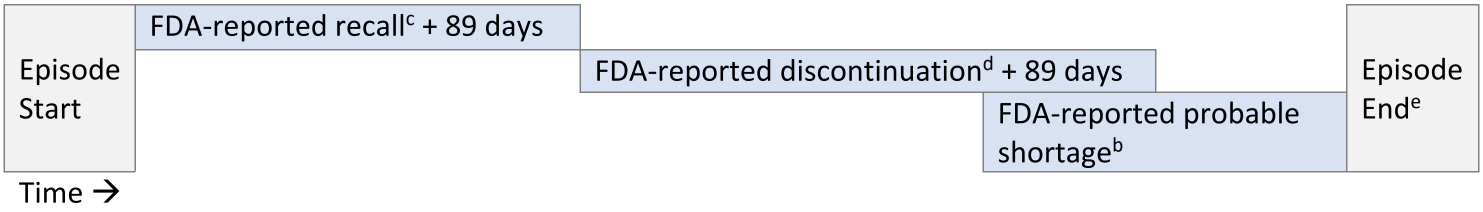

Color Key	
(blue)	Indicates months with any active FDA or ASHP supply issue
(gray)	Indicates assigned starting or ending date of active supply issue
(white)	Months with no active reports

© 2024 Callaway Kim K et al. *JAMA Network Open.*

ASHP, American Society of Health System Pharmacists; FDA, Food and Drug Administration; CDER, Center for Drug Evaluation and Research; CBER, Center for Biologics Evaluation and Research

a. ASHP shortage reports were sourced from the society's public website (https://www.ashp.org/drug-shortages/current-shortages?loginreturnUrl=SSOCheckOnly). Start dates were assigned using the "Created" date at the bottom of each report. For resolved shortages, end dates were assigned using the most recent "Updated" date at the bottom of each report.
b. FDA shortage reports were sourced from the CDER (https://www.accessdata.fda.gov/scripts/drugshortages/default.cfm) and CBER (https://www.fda.gov/vaccines-blood-biologics/safety-availability-biologics/cber-regulated-products-current-shortages) public websites. For reports prior to November 2021, data were downloaded using the Wayback Machine Internet Archive (https://archive.org/web). Start dates were assigned using the "Initial Posting Date" variable. For resolved shortages, end dates were assigned using the date of the first data download for which that listing had a "Status" equal to "Resolved". For dates with no available data on the Internet Archive, ending dates were assigned using the mid-point of the missing date range. For example, if a shortage was listed as "Status" equal to "Current" on 1/1/2019 and "Status" equal to "Resolved" on 1/10/2019 with no available archived records between these dates, the end date of the shortage was assigned to the mid-point (1/5/2019).
c. FDA recall reports were sourced from the CDER (https://www.fda.gov/safety/recalls-market-withdrawals-safety-alerts) and CBER (https://www.fda.gov/vaccines-blood-biologics/safety-availability-biologics/recalls-biologics) public websites. Starting dates were assigned using the "Date" variable. Recalls were terminated after 90 days.
d. Discontinuations were identified as records with "Status" equal to "To be discontinued" within the FDA CDER database (https://www.accessdata.fda.gov/scripts/drugshortages/default.cfm). Starting dates were assigned using the "Initial Posting Date" variable. Discontinuations were terminated after 90 days.
e. Reports which had not been resolved were censored at the end of the study period (December 2021).

© 2024 Callaway Kim K et al. *JAMA Network Open.*

e-Figure 3: Examples of Medications with No Supply Decrease, Meaningful (≥33%) Shortage, and Severe (≥66%) Shortage in MIDAS

e-Figure 3 provides three examples of supply chain issue reports and matched, unexposed comparison drugs which (A) were not associated with a drug shortage (≥33% decrease in purchased units) within 6 months, (B) were associated with a meaningful drug shortage (≥33% decrease) and (C) were associated with a severe drug shortage (≥66% decrease).

A. Example of Supply Chain Issue Resulting in No Supply Decrease in MIDAS[a]

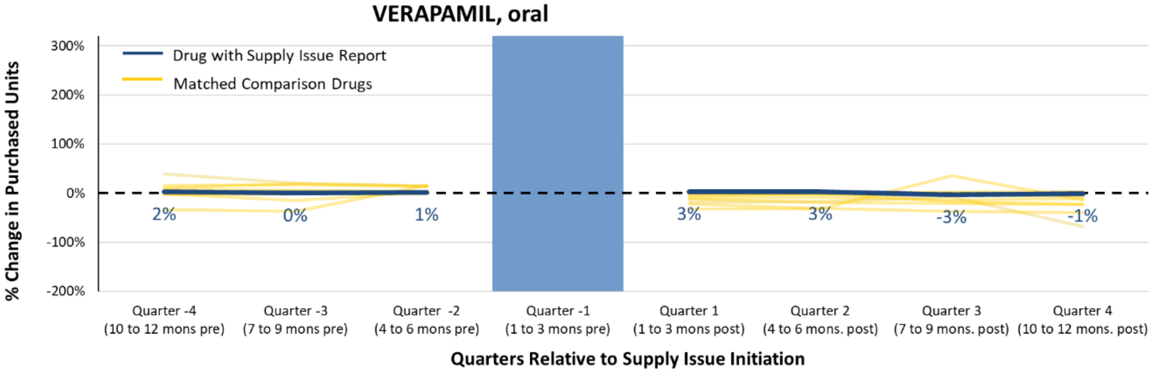

B. Example of Supply Chain Issue Resulting in a Meaningful Shortage (≥33% Supply Decrease) in MIDAS[b]

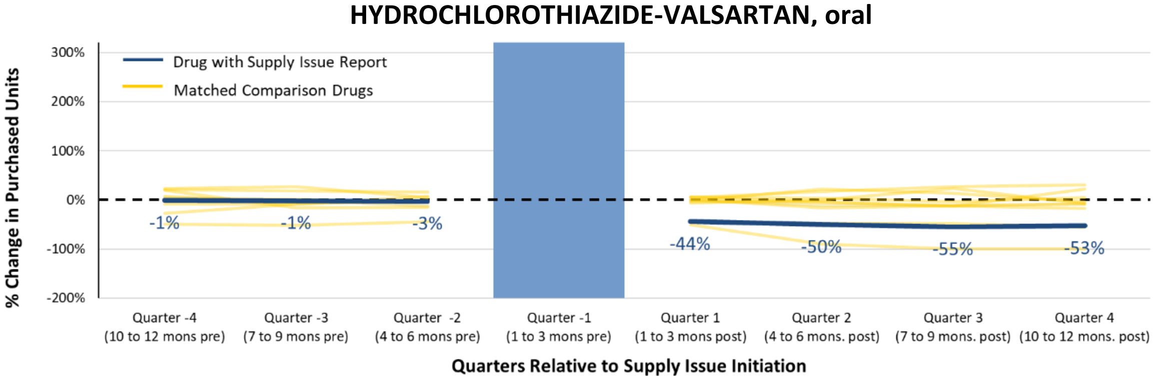

C. Example of Supply Chain Issue Resulting in a Severe Shortage (≥66% Supply Decrease) in MIDAS[c]

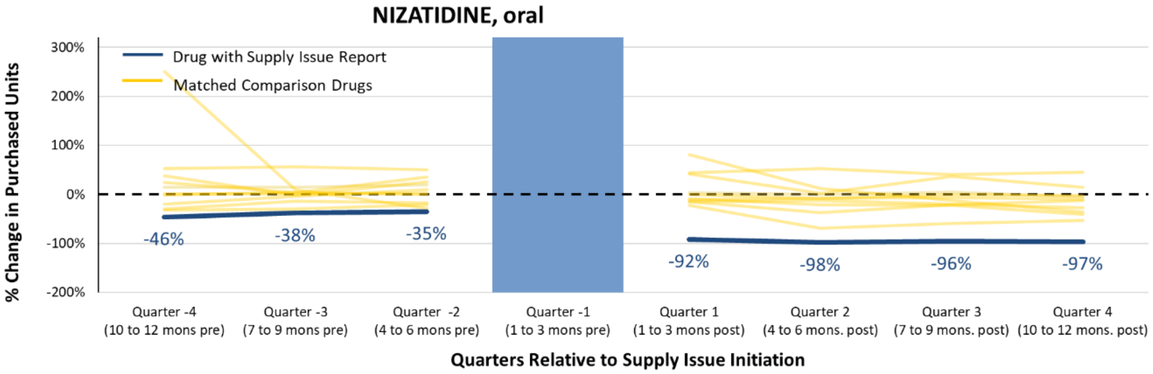

Abbreviations: mons= months, Units represent the smallest level of consumption (e.g.: 1 pill, 1 vial/ampule, or 5mL of liquid). Percentage changes are relative to Quarter -1 (1 to 3 months pre-supply-chain-issue report)

© 2024 Callaway Kim K et al. *JAMA Network Open.*

a. Example was for a discontinuation of VERAPAMIL reported by both FDA and ASHP which began in February 2019. Matched comparison drugs were PROBENECID (oral), AMOXICILLIN-CLARITHROMYCIN-LANSOPRAZOLE (oral), LINAGLIPTIN (oral), MESALAZINE (oral), RITUXIMAB (parenteral), RISPERIDONE (parenteral), TELAVANCIN (parenteral), AFLIBERCEPT (parenteral), ACETYLSALICYLIC ACID-CARISOPRODOL (oral), and VACCINE, PNEUMOCOCCAL (parenteral).
b. Example was for a recall and subsequent shortage of HYDROCHLOROTHIAZIDE-VALSARTAN due to nitrosamine impurities which began in July 2018 and was reported by both FDA and ASHP. Matched comparison drugs were FORMOTEROL-MOMETASONE (inhaled), MECASERMIN (parenteral), CIPROFLOXACIN-DEXAMETHASONE (otic), FOSCARNET SODIUM (parenteral), LEVOCARNITINE (oral), ROMIDEPSIN (parenteral), CETIRIZINE-PSEUDOEPHEDRINE (oral), PHENYLEPHRINE (ophthalmic), BLINATUMOMAB (parenteral) and FLUNISOLIDE (inhaled)
c. Example was for a shortage of NIZATIDINE reported by both FDA and ASHP which began in January 2020. Matched comparison drugs were NALOXONE (parenteral), DAUNORUBICIN (parenteral), LINCOMYCIN (parenteral), VORICONAZOLE (parenteral), IMMUNOGLOBULIN ANTI-HEPATITIS B SURFACE ANTIGEN (parenteral), IMIGLUCERASE (parenteral), AMLODIPINE-TELMISARTAN (oral), GRAMICIDIN-NEOMYCIN-POLYMYXIN B (ophthalmic), FLAVOXATE (oral), and MIFEPRISTONE (oral).

© 2024 Callaway Kim K et al. *JAMA Network Open.*

e-Table 2: Covariate Definitions

e-Table 2 provides detailed definitions for each covariate used in our random effects logistic regression models.

Variable	Definition
Formulation	Use New Form Code (NFC123) variable in MIDAS to assign drugs into the following formulation categories:<br>• *Parenteral*: NFC123 codes starting with "F" or "G".<br>• *Oral*: NFC123 codes starting with "A", "B", or "D", in addition to certain systemic liquids ("EEK", "EEP", "EGA", "EGM", "JGA", "JGE", "JRP", "JVA", "JVN", "JVP"). Oral topical products ("ACS", "ASB", "AYG", "DEH", and "DEK") were excluded.<br>• *Ophthalmic and otic*: NFC123 codes starting with "N" or "P".<br>• *Inhaled*: NFC123 codes starting with "R" or "Q", in addition to certain inhaled liquids ("JCT", "JEN", and "JEY").
Number of manufacturers	Calculated in the 6-month period prior to each drug's index date using the manufacturer variable in MIDAS. "REPACKAGER" was counted as one manufacturer.
WHO essential medicine status	Assigned using the agency's full electronic eEML database, downloaded in June 2022. The following drugs were considered essential medicines based on the molecule list variable in MIDAS and above formulation categories:<br>• *Oral essential medicines*: "ABACAVIR", "ABACAVIR!LAMIVUDINE", "ABEMACICLIB", "ABIRATERONE ACETATE", "ACETAZOLAMIDE", "ACETYLSALICYLIC ACID", "AFATINIB", "ALBENDAZOLE", "ALLOPURINOL", "AMIKACIN", "AMILORIDE", "AMINOSALICYLIC ACID", "AMITRIPTYLINE", "AMLODIPINE", "AMLODIPINE!TELMISARTAN", "AMOXICILLIN", "AMOXICILLIN!CLAVULANIC ACID", "ANASTROZOLE", "APREPITANT", "ARTEMETHER!LUMEFANTRINE", "ASCORBIC ACID", "ATAZANAVIR", "ATENOLOL", "ATORVASTATIN", "AZATHIOPRINE", "AZITHROMYCIN", "BARIUM", "BEDAQUILINE", "BENSERAZIDE!LEVODOPA", "BICALUTAMIDE", "BINIMETINIB", "BISACODYL", "BISOPROLOL", "BROMOCRIPTINE", "BUMETANIDE", "BUPRENORPHINE", "BUPROPION", "CABERGOLINE", "CALCITRIOL", "CALCIUM", "CALFACTANT", "CANAGLIFLOZIN", "CAPECITABINE", "CAPTOPRIL", "CARBAMAZEPINE", "CARBIDOPA!LEVODOPA", "CARVEDILOL", "CEFALEXIN", "CEFAZOLIN", "CEFAZOLIN!GLUCOSE", "CEFIXIME", "CEFOTAXIME", "CEFTAZIDIME", "CEFTRIAXONE", "CEFTRIAXONE!GLUCOSE", "CETIRIZINE", "CHLORAMBUCIL", "CHLOROQUINE", "CHLOROTHIAZIDE", "CHLORPROMAZINE", "CHLORTALIDONE", "CIMETIDINE", "CIPROFLOXACIN", "CITALOPRAM", "CLARITHROMYCIN", "CLINDAMYCIN", "CLOFAZIMINE", "CLOMIFENE", "CLOMIPRAMINE", "CLONAZEPAM", "CLOPIDOGREL", "CLOXACILLIN", "CLOZAPINE", "COBICISTAT!ELVITEGRAVIR!EMTRICITABINE!TENOFOVIR ALAFENAMIDE", "COBICISTAT!ELVITEGRAVIR!EMTRICITABINE!TENOFOVIR DISOPROXIL", "COBIMETINIB", "CODEINE", "COLCHICINE", "COLECALCIFEROL", "CRIZOTINIB", "CYCLIZINE", "CYCLOPHOSPHAMIDE", "CYCLOSERINE", "DABIGATRAN ETEXILATE", "DABRAFENIB", "DACLATASVIR", "DAPAGLIFLOZIN", "DAPSONE", "DARUNAVIR", "DASABUVIR", "DASATINIB", "DEFERASIROX", "DEXAMETHASONE", "DIAZEPAM", "DIAZOXIDE", "DIDANOSINE", "DIHYDROERGOTOXINE", "DOCUSATE", "DOLASETRON", "DOLUTEGRAVIR", "DOLUTEGRAVIR!LAMIVUDINE", "DOXYCYCLINE", "EDOXABAN", "EFAVIRENZ", "EFAVIRENZ!EMTRICITABINE!TENOFOVIR DISOPROXIL", "EFAVIRENZ!LAMIVUDINE!TENOFOVIR DISOPROXIL", "ELBASVIR!GRAZOPREVIR", "EMPAGLIFLOZIN", "EMTRICITABINE", "EMTRICITABINE!RILPIVIRINE!TENOFOVIR ALAFENAMIDE", "EMTRICITABINE!RILPIVIRINE!TENOFOVIR DISOPROXIL", "EMTRICITABINE!TENOFOVIR ALAFENAMIDE", "EMTRICITABINE!TENOFOVIR DISOPROXIL", "ENALAPRIL", "ENCORAFENIB", "ENTECAVIR", "ENZALUTAMIDE", "EPHEDRINE", "ERGOCALCIFEROL", "ERLOTINIB", "ESCITALOPRAM", "ETHAMBUTOL", "ETHINYLESTRADIOL!LEVONORGESTREL", "ETHINYLESTRADIOL!NORETHISTERONE", "ETHIONAMIDE", "ETHOSUXIMIDE",

© 2024 Callaway Kim K et al. *JAMA Network Open.*

“EVEROLIMUS”, “FEXOFENADINE”, “FINGOLIMOD”, “FLUDROCORTISONE”, “FLUOXETINE”, “FLUTAMIDE”, “FLUVASTATIN”, “FLUVOXAMINE”, “FOLIC ACID”, “FOLIC ACID!IRON FERROUS”, “GABAPENTIN”, “GATIFLOXACIN”, “GEFITINIB”, “GENTAMICIN”, “GENTAMICIN!SODIUM”, “GLECAPREVIR!PIBRENTASVIR”, “GLIBENCLAMIDE”, “GLICLAZIDE”, “GLUCOSE!POTASSIUM!SODIUM”, “GLUCOSE!POTASSIUM!SODIUM!ZINC”, “GRANISETRON”, “GRISEOFULVIN”, “HALOPERIDOL”, “HYDROCHLOROTHIAZIDE”, “HYDROCHLOROTHIAZIDE!LISINOPRIL”, “HYDROCHLOROTHIAZIDE!TELMISARTAN”, “HYDROCORTISONE”, “HYDROMORPHONE”, “HYDROXYCARBAMIDE”, “HYDROXYCHLOROQUINE”, “IBRUTINIB”, “IBUPROFEN”, “IMATINIB”, “INDAPAMIDE”, “INDINAVIR”, “INDOMETACIN”, “IODINE”, “IRON FERROUS”, “ISONIAZID”, “ISONIAZID!PYRAZINAMIDE!RIFAMPICIN”, “ISONIAZID!RIFAMPICIN”, “ISOSORBIDE DINITRATE”, “ITRACONAZOLE”, “IVERMECTIN”, “LACTULOSE”, “LAMIVUDINE”, “LAMIVUDINE!TENOFOVIR DISOPROXIL”, “LAMIVUDINE!ZIDOVUDINE”, “LAMOTRIGINE”, “LEDIPASVIR!SOFOSBUVIR”, “LENALIDOMIDE”, “LEVODOPA”, “LEVOFLOXACIN”, “LEVONORGESTREL”, “LEVOTHYROXINE SODIUM”, “LINEZOLID”, “LISINOPRIL”, “LITHIUM”, “LOPERAMIDE”, “LOPINAVIR!RITONAVIR”, “LORATADINE”, “LOSARTAN”, “LOVASTATIN”, “MEBENDAZOLE”, “MEDROXYPROGESTERONE”, “MEFLOQUINE”, “MERCAPTOPURINE”, “MESALAZINE”, “METFORMIN”, “METHADONE”, “METHIONINE”, “METHOTREXATE”, “METHYLDOPA”, “METHYLERGOMETRINE”, “METHYLPHENIDATE”, “METOCLOPRAMIDE”, “METOPROLOL”, “METRONIDAZOLE”, “MIDAZOLAM”, “MIFEPRISTONE!MISOPROSTOL”, “MILTEFOSINE”, “MISOPROSTOL”, “MORPHINE”, “MOXIFLOXACIN”, “NELFINAVIR”, “NEOSTIGMINE”, “NEVIRAPINE”, “NICOTINAMIDE”, “NICOTINE”, “NIFEDIPINE”, “NIFURTIMOX”, “NILOTINIB”, “NILUTAMIDE”, “NITROFURANTOIN”, “NITROGLYCERIN”, “NORETHISTERONE”, “NYSTATIN”, “OFLOXACIN”, “OMBITASVIR!PARITAPREVIR!RITONAVIR”, “OMEPRAZOLE”, “OSELTAMIVIR”, “OSIMERTINIB”, “OXYCODONE”, “PALBOCICLIB”, “PALONOSETRON”, “PANCRELIPASE”, “PARACETAMOL”, “PAROMOMYCIN”, “PAROXETINE”, “PENICILLAMINE”, “PENICILLIN V”, “PENTAMIDINE”, “PHENOBARBITAL”, “PHENYTOIN”, “PIPERACILLIN!TAZOBACTAM”, “POTASSIUM”, “PRAMIPEXOLE”, “PRAVASTATIN”, “PRAZIQUANTEL”, “PREDNISOLONE”, “PREDNISONE”, “PRIMAQUINE”, “PROBENECID”, “PROCARBAZINE”, “PROMETHAZINE”, “PROPRANOLOL”, “PROPYLTHIOURACIL”, “PYRANTEL”, “PYRAZINAMIDE”, “PYRIDOXINE”, “PYRIMETHAMINE”, “QUINIDINE”, “RALTEGRAVIR”, “RANITIDINE”, “RIBOCICLIB”, “RIBOFLAVIN”, “RIFABUTIN”, “RIFAMPICIN”, “RIFAPENTINE”, “RISPERIDONE”, “RITONAVIR”, “RIVAROXABAN”, “ROPINIROLE”, “SAQUINAVIR”, “SENNA”, “SERTRALINE”, “SIMEPREVIR”, “SIMVASTATIN”, “SOFOSBUVIR”, “SOFOSBUVIR!VELPATASVIR”, “SPIRONOLACTONE”, “STAVUDINE”, “SUCCIMER”, “SULFADIAZINE”, “SULFAMETHOXAZOLE!TRIMETHOPRIM”, “SULFASALAZINE”, “SUMATRIPTAN”, “TAMOXIFEN”, “TEDIZOLID”, “TENOFOVIR ALAFENAMIDE”, “TENOFOVIR DISOPROXIL”, “TERBUTALINE”, “TETRACYCLINE”, “THALIDOMIDE”, “THIAMAZOLE”, “THIAMINE”, “TINIDAZOLE”, “TIOGUANINE”, “TOLBUTAMIDE”, “TORASEMIDE”, “TRAMADOL”, “TRAMETINIB”, “TRETINOIN”, “TRIHEXYPHENIDYL”, “TRIMETHOPRIM”, “ULIPRISTAL ACETATE”, “VALACICLOVIR”, “VALGANCICLOVIR”, “VALPROIC ACID”, “VANCOMYCIN”, “VARENICLINE”, “VEMURAFENIB”, “VERAPAMIL”, “WARFARIN”, “ZANUBRUTINIB”, “ZINC”

- *Parenteral essential medicines*: “ACETYLCYSTEINE”, “ACICLOVIR”, “ADALIMUMAB”, “ALPROSTADIL”, “ALTEPLASE”, “AMIKACIN”, “AMIODARONE”, “AMOXICILLIN”, “AMOXICILLIN!CLAVULANIC ACID”, “AMPHOTERICIN B”, “AMPICILLIN”, “ANAKINRA”, “ANIDULAFUNGIN”, “APIXABAN”, “ARSENIC”, “ARTESUNATE”, “ATEZOLIZUMAB”, “ATRACURIUM BESILATE”, “ATROPINE”, “AVIBACTAM!CEFTAZIDIME”, “AZACITIDINE”, “AZATHIOPRINE”, “AZITHROMYCIN”, “AZTREONAM”, “BENDAMUSTINE”, “BLEOMYCIN”, “BORTEZOMIB”, “BUMETANIDE”, “BUPIVACAINE”, “BUPRENORPHINE”, “C1 INHIBITOR (HUMAN)”, “CAFFEINE”, “CALCITRIOL”, “CALCIUM”, “CAPREOMYCIN”, “CARBETOCIN”, “CARBOPLATIN”, “CASPOFUNGIN”, “CEFAZOLIN”, “CEFEPIME”, “CEFIDEROCOL”, “CEFOTAXIME”, “CEFTAROLINE FOSAMIL”, “CEFTAZIDIME”, “CEFTOLOZANE!TAZOBACTAM”, “CEFTRIAXONE”, “CEFTRIAXONE!GLUCOSE”, “CEFUROXIME”, “CERTOLIZUMAB PEGOL”, “CETIRIZINE”, “CHLORAMPHENICOL”, “CHLOROQUINE”, “CHLOROTHIAZIDE”, “CICLOSPORIN”, “CILASTATIN!IMIPENEM”, “CIPROFLOXACIN”, “CLARITHROMYCIN”, “CLARITHYOMYCIN”, “CLINDAMYCIN”, “CLOXACILLIN”, “COLISTIN”, “CYANOCOBALAMIN”, “CYTARABINE”, “DACARBAZINE”, “DACTINOMYCIN”, “DALTEPARIN SODIUM”, “DAPTOMYCIN”, “DARATUMUMAB”, “DARBEPOETIN ALFA”, “DAUNORUBICIN”, “DEFEROXAMINE”, “DELAFLOXACIN”, “DESMOPRESSIN”, “DEXAMETHASONE”, “DEXTRAN”, “DIGOXIN”, “DINOPROSTONE”, “DOCETAXEL”, “DOLASETRON”, “DOPAMINE”, “DOXORUBICIN”, “DURVALUMAB”, “EFLORNITHINE”, “ENOXAPARIN SODIUM”, “EPHEDRINE”, “EPINEPHRINE”, “EPINEPHRINE!LIDOCAINE”, “EPOETIN ALFA”, “EPOETIN ZETA”, “ERAVACYCLINE”, “ERGOMETRINE”, “ERYTHROMYCIN”, “ETANERCEPT”, “ETONOGESTREL”, “ETOPOSIDE”, “FACTOR IX”, “FACTOR VIII”, “FILGRASTIM”,

© 2024 Callaway Kim K et al. *JAMA Network Open.*

“FLUCONAZOLE”, “FLUCYTOSINE”, “FLUDARABINE”, “FLUOROURACIL”, “FLUPHENAZINE”, “FOMEPIZOLE”, “FOSFOMYCIN (INJECTION)”, “FULVESTRANT”, “FUROSEMIDE”, “GEMCITABINE”, “GENTAMICIN”, “GENTAMICIN!SODIUM”, “GLATIRAMER ACETATE”, “GLUCAGON”, “GLUCOSE”, “GLUCOSE!SODIUM”, “GLYCOPYRRONIUM”, “GOLIMUMAB”, “GOSERELIN”, “GRANISETRON”, “HALOPERIDOL”, “HEPARIN!SODIUM”, “HYDRALAZINE”, “HYDROCORTISONE”, “HYDROMORPHONE”, “HYDROXOCOBALAMIN”, “IBUPROFEN”, “IFOSFAMIDE”, “IMMUNOGLOBULIN ANTI-CLOSTRIDIUM TETANI TOXIN”, “IMMUNOGLOBULIN ANTI-D (RHO)”, “IMMUNOGLOBULIN ANTI-RABIES”, “IMMUNOGLOBULIN ANTIVENOM CROTALIDAE SNAKES”,
“IMMUNOGLOBULIN ANTIVENOM LATRODECTUS MACTANS”, “IMMUNOGLOBULIN ANTIVENOM MICRURUS FULVIUS”, “IMMUNOGLOBULIN BASE”, “IMMUNOGLOBULIN, ANTI-RABIES”, “INDOMETACIN”, “INFLIXIMAB”, “INSULIN ASPART”, “INSULIN ASPART!INSULIN ASPART PROTAMINE”, “INSULIN ASPART!INSULIN ASPART PROTAMINE CRYSTALLINE”, “INSULIN DEGLUDEC”, “INSULIN DEGLUDEC!LIRAGLUTIDE”, “INSULIN DETEMIR”, “INSULIN GLARGINE”, “INSULIN GLARGINE!LIXISENATIDE”, “INSULIN GLULISINE”, “INSULIN HUMAN BASE”, “INSULIN HUMAN BASE!INSULIN HUMAN ISOPHANE”, “INSULIN HUMAN ISOPHANE”, “INSULIN LISPRO”, “INSULIN LISPRO!INSULIN LISPRO PROTAMINE”, “INSULIN PROCINE BASE”, “INSULIN PROCINE ISOPHANE”, “INTERFERON ALFA-2B”, “IODINE”, “IOHEXOL”, “IRINOTECAN”, “ISOPRENALINE”, “KETAMINE”, “LEUPRORELIN”, “LEVONORGESTREL”, “LIDOCAINE”, “LINEZOLID”, “LORAZEPAM”, “MAGNESIUM”, “MANNITOL”, “MEDROXYPROGESTERONE”, “MELPHALAN”, “MEROPENEM”, “MEROPENEM!VABORBACTAM”, “MESNA”, “METHOTREXATE”, “METHOXY POLYETHYLENE GLYCOL-EPOETIN BETA”, “METHYLERGOMETRINE”,“METHYLPREDNISOLONE”, “METHYLTHIONINIUM”, “METOPROLOL”, “METRONIDAZOLE”, “MICAFUNGIN”, “MIDAZOLAM”, “MORPHINE”, “NADROPARIN CALCIUM”, “NALOXONE”, “NIVOLUMAB”, “NORETHISTERONE”, “OMADACYCLINE”, “ONDANSETRON”, “OXALIPLATIN”, “OXYTOCIN”, “PACLITAXEL”, “PALIPERIDONE”, “PALONOSETRON”, “PAROMOMYCIN”, “PEGASPARGASE”, “PEGINTERFERON ALFA-2A”, “PEMBROLIZUMAB”, “PENICILLIN G”, “PENTAMIDINE”, “PERTUZUMAB”, “PHYTOMENADIONE”, “PIPERACILLIN!TAZOBACTAM”, “PLAZOMICIN”, “POLYMYXIN B”, “POTASSIUM”, “PRALIDOXIME”, “PROCAINAMIDE”, “PROPOFOL”, “PROPRANOLOL”, “PROTAMINE”, “PYRIDOSTIGMINE”, “QUININE”, “RASBURICASE”, “RETINOL”, “RIBAVIRIN”, “RITUXIMAB”, “SALBUTAMOL”, “SODIUM”, “STREPTOMYCIN”, “SULFAMETHOXAZOLE!TRIMETHOPRIM”, “SUXAMETHONIUM”, “TACROLIMUS”, “TEDIZOLID”, “TERBUTALINE”, “TESTOSTERONE”, “TIGECYCLINE”, “TOBRAMYCIN”, “TOCILIZUMAB”, “TRANEXAMIC ACID”, “TRASTUZUMAB”, “TRASTUZUMAB EMTANSINE”, “TRIAMCINOLONE ACETONIDE”, “TRIAMCINOLONE HEXACETONIDE”, “TRIPTORELIN”, “TUBERCULIN TEST”, “VACCINE, ACELL.PERT.TET.& DIP.”, “VACCINE, BACILLUS CALMETTE-GUERIN”, “VACCINE, CHOLERA”, “VACCINE, DIPHTHERIA AND TETANUS”, “VACCINE, HAEMOPHILUS B CONJUCATE”, “VACCINE, HEP.A INACTIV. VIRUS”, “VACCINE, HEPATITIS B”, “VACCINE, HEPATITIS B SURFACE ANTIGEN”, “VACCINE, HUMAN PAPILLOMAVIRUS (HPV) TYPE-16 & 18”, “VACCINE, HUMAN PAPILLOMAVIRUS (HPV) TYPE-6,11,16 & 18”, “VACCINE, HUMAN PAPILLOMAVIRUS (HPV) TYPE-6,11,16,18,31,33,45,52,58”, “VACCINE, INFLUENZA”, “VACCINE, JAPAN.ENCEPH.VIR.SA14-14-2”, “VACCINE, PNEUMOCOCCAL”, “VACCINE, PNEUMOCOCCAL CONJUGATE”, “VACCINE, POLIOMYELITIS”, “VACCINE, RABIES”, “VACCINE, ROTAVIRUS”, “VACCINE, TICK BORNE ENCEPHALITIS”, “VACCINE, TYPHOID”, “VACCINE, VARICELLA ZOSTER”, “VACCINE, YELLOW FEVER LIVE”, “VACCINE,MENINGOCOCCAL C CONJUGATE”, “VACCINE,MENINGOCOCCAL CONJUGATE”, “VACCINE,MENINGOCOCCAL POLYSACCHARIDE”, “VANCOMYCIN”, “VECURONIUM BROMIDE”, “VERAPAMIL”, “VINBLASTINE”, “VINCRISTINE”, “VINORELBINE”, “VORICONAZOLE”, “WATER FOR INJECTION/INFUSION”, “ZIDOVUDINE”, “ZOLEDRONIC ACID”

- *Ophthalmic and otic essential medicines*: “ACICLOVIR”, “ATROPINE”, “AZITHROMYCIN”, “BEVACIZUMAB”, “CARBACHOL”, “CETIRIZINE”, “CLOTRIMAZOLE”, “CYCLOPENTOLATE”, “DEXTRAN”, “EPINEPHRINE”, “ERYTHROMYCIN”, “FLUORESCEIN”, “GENTAMICIN”, “HOMATROPINE”, “HYDROCORTISONE”, “LATANOPROST”, “NATAMYCIN”, “OFLOXACIN”, “PILOCARPINE”, “PREDNISOLONE”, “RANIBIZUMAB”, “SILVER”, “SULFACETAMIDE”, “TETRACAINE”, “TETRACYCLINE”, “TIMOLOL”, “TOBRAMYCIN”, “TRIAMCINOLONE ACETONIDE”, “TROPICAMIDE”
- *Inhaled essential medicines*: “ACLIDINIUM BROMIDE”, “BECLOMETASONE”, “BERACTANT”, “BUDESONIDE”, “BUDESONIDE!FORMOTEROL”, “CICLESONIDE”, “FLUNISOLIDE”, “FLUTICASONE”, “FLUTICASONE FUROATE!VILANTEROL”, “FORMOTEROL”, “FORMOTEROL!MOMETASONE”,

© 2024 Callaway Kim K et al. *JAMA Network Open.*

	"IPRATROPIUM BROMIDE", "ISOFLURANE", "MOMETASONE", "PORACTANT ALFA", "SALMETEROL", "TERBUTALINE", "TIOTROPIUM BROMIDE", "TOBRAMYCIN", "UMECLIDINIUM BROMIDE"
Provider-administered drugs	Provider administered drugs were identified as those with a HCPC "J" procedure code typically administered by clinicians to diagnose or manage diseases, that required internal device placement (e.g., IUD, implants, insulin pumps) and/or that were IV products. Drugs administered by patients for chronic disease management (e.g., steroid inhalers) were excluded.<br>• *Inhaled provider-administered drugs*: "ACETYLCYSTEINE", "MANNITOL", "METHACHOLINE", "PENTAMIDINE", "REVEFENACIN"<br>• *Ophthalmic provider-administered drugs*: "DEXAMETHASONE", "KETOROLAC!PHENYLEPHRINE", "MITOMYCIN", "RIBOFLAVIN"<br>• *Parenteral provider-administered drugs*: "ABATACEPT", "ABCIXIMAB", "ACEPROMAZINE", "ACETAZOLAMIDE", "ACETYLCYSTEINE", "ACICLOVIR", "ADALIMUMAB", "ADENOSINE", "ADUCANUMAB", "AFLIBERCEPT", "AGALSIDASE BETA", "ALBUTREPENONACOG ALFA" , "ALDESLEUKIN", "ALEMTUZUMAB", "ALGLUCOSIDASE ALFA", "ALPROSTADIL", "ALTEPLASE", "AMIFOSTINE", "AMIKACIN", "AMINOPHYLLINE", "AMIODARONE", "AMITRIPTYLINE", "AMIVANTAMAB", "AMOBARBITAL", "AMPHOTERICIN B", "AMPICILLIN", "AMPICILLIN!SULBACTAM", "ANDEXANET ALFA", "ANIDULAFUNGIN", "ANIFROLUMAB", "ANTITHROMBIN ALFA", "ANTITHROMBIN III", "APOMORPHINE", "APREPITANT", "APROTININ!CALCIUM!FIBRINOGEN!THROMBIN", "ARGATROBAN", "ARIPIPRAZOLE", "ARSENIC", "ASPARAGINASE ERWINIA CHRYSANTHEMI", "ASPARAGINASE ESCHERICHIA COLI", "ATEZOLIZUMAB", "ATROPINE", "AUROTHIOMALIC ACID", "AVELUMAB", "AVIBACTAM!CEFTAZIDIME", "AZACITIDINE", "AZATHIOPRINE", "AZITHROMYCIN", "BACLOFEN", "BASILIXIMAB", "BELANTAMAB", "BELATACEPT", "BELIMUMAB", "BELINOSTAT", "BENDAMUSTINE", "BENRALIZUMAB", "BENZATROPINE", "BETAMETHASONE", "BETHANECHOL", "BEVACIZUMAB", "BEZLOTOXUMAB", "BIMATOPROST", "BIVALIRUDIN", "BLEOMYCIN", "BLINATUMOMAB", "BORTEZOMIB", "BRENTUXIMAB VEDOTIN", "BREXANOLONE", "BROLUCIZUMAB", "BROMPHENIRAMINE", "BUPRENORPHINE", "BUROSUMAB", "BUSULFAN", "BUTORPHANOL", "C1 INHIBITOR (HUMAN) ", "CABAZITAXEL", "CABOTEGRAVIR", "CABOTEGRAVIR!RILPIVIRINE", "CAFFEINE", "CALASPARGASE PEGOL", "CALCITONIN SALMON", "CALCITRIOL", "CALCIUM", "CALCIUM FOLINATE", "CALCIUM LEVOFOLINATE", "CALCIUM!DL-LACTIC ACID!POTASSIUM!SODIUM", "CALCIUM!POTASSIUM!SODIUM", "CANAKINUMAB", "CARBOPLATIN", "CARFILZOMIB", "CARMUSTINE", "CASIMERSEN", "CASPOFUNGIN", "CEFAZOLIN", "CEFEPIME", "CEFIDEROCOL", "CEFOTAXIME", "CEFOXITIN", "CEFTAROLINE FOSAMIL", "CEFTAZIDIME", "CEFTOLOZANE!TAZOBACTAM", "CEFTRIAXONE", "CEFUROXIME", "CEMIPLIMAB", "CERLIPONASE ALFA", "CERTOLIZUMAB PEGOL", "CETIRIZINE", "CETUXIMAB", "CHLORAMPHENICOL", "CHLORDIAZEPOXIDE", "CHLORMETHINE", "CHLOROPROCAINE", "CHLOROQUINE", "CHLOROTHIAZIDE", "CHLORPROMAZINE", "CHORIONIC GONADOTROPIN", "CICLOSPORIN", "CIDOFOVIR", "CILASTATIN!IMIPENEM", "CILASTATIN!IMIPENEM!RELEBACTAM", "CIPROFLOXACIN", "CISPLATIN", "CLADRIBINE", "CLOFARABINE", "CLONIDINE", "CLOSTRIDIUM BOTULINUM TOXIN TYPE A", "CODEINE", "COLCHICINE", "COLISTIN", "COLLAGENASE CLOSTRIDIUM HISTOLYTICUM", "COPANLISIB", "CORTICORELIN", "CORTICOTROPIN", "CRIZANLIZUMAB", "CYANOCOBALAMIN", "CYCLOPHOSPHAMIDE", "CYTARABINE", "CYTARABINE!DAUNORUBICIN", "DACLIZUMAB", "DACTINOMYCIN", "DALBAVANCIN", "DALFOPRISTIN!QUINUPRISTIN", "DAPTOMYCIN", "DARATUMUMAB", "DARATUMUMAB!VORHYALURONIDASE", "DARBEPOETIN ALFA", "DAUNORUBICIN", "DECITABINE", "DEFEROXAMINE", "DEGARELIX", "DENOSUMAB", "DEOXYCHOLIC ACID", "DESMOPRESSIN", "DEXAMETHASONE", "DEXRAZOXANE", "DEXTRAN", "DIAZEPAM, "DIAZOXIDE", "DICLOFENAC", "DICYCLOVERINE", "DIETHYLSTILBESTROL", "DIGOXIN", "DIGOXIN SPECIFIC ANTIBODY FRAGMENTS", "DIHYDROERGOTAMINE", "DIMENHYDRINATE", "DIMETHYL SULFOXIDE", "DIPHENHYDRAMINE", "DIPYRIDAMOLE", "DOBUTAMINE",

© 2024 Callaway Kim K et al. *JAMA Network Open.*

“DOCETAXEL”, “DOLASETRON”, “DOPAMINE”, “DORIPENEM”, “DOSTARLIMAB”, “DOXERCALCIFEROL”, “DOXORUBICIN”, “DROPERIDOL”, “DURVALUMAB”, “ECALLANTIDE”, “ECULIZUMAB”, “EDARAVONE”, “EDETIC ACID”, “EFTRENONACOG ALFA”, “ELOSULFASE ALFA”, “ELOTUZUMAB”, “EMAPALUMAB”, “EMICIZUMAB”, “ENFORTUMAB VEDOTIN”, “ENFUVIRTIDE”, “EPINEPHRINE”, “EPIRUBICIN”, “EPOETIN ALFA”, “EPOPROSTENOL”, “EPTIFIBATIDE”, “EPTINEZUMAB”, “ERAVACYCLINE”, “ERGOMETRINE”, “ERIBULIN”, “ERTAPENEM”, “ERYTHROMYCIN”, “ESTRADIOL”, “ESTROGENIC SUBSTANCES, CONJUGATED”, “ESTRONE”, “ETANERCEPT”, “ETELCALCETIDE”, “ETEPLIRSEN”, “ETOPOSIDE”, “EVINACUMAB”, “FACTOR IX”, “FACTOR IX!FACTOR VII!FACTOR X!PROTEIN C!PROTEIN S!PROTHROMBIN”, “FACTOR IX!FACTOR VII!FACTORX!PROTEIN C!PROETIN S!PROTHROMBIN”, “FACTOR VIII”, “FACTOR VIII!FACTOR VON WILLEBRAND”, “FACTOR X”, “FACTOR XIII”, “FENTANYL”, “FIBRINOGEN”, “FILOGRASTIM”, “FLOXURIDINE”, “FLUCONAZOLE”, “FLUDARABINE”, “FLUOCINOLONE ACETONIDE”, “FLUOROURACIL”, “FLUPHENAZINE”, “FOMEPIZOLE”, “FONDAPARINUX SODIUM”, “FOSAPREPITANT”, “FOSCARNET SODIUM”, “FOSNETUPITANT!PALONOSET”, “FREMANEZUMAB”, “FULVESTRANT”, “FUROSEMIDE”, “GALSULFASE”, “GANCICLOVIR”, “GATIFLOXACIN”, “GEMCITABINE”, “GEMTUZUMAB OZOGAMICIN”, “GENTAMICIN”, “GIVOSIRAN”, “GLATIRAMER ACETATE”, “GLUCAGON”, “GLUCOSE”, “GLUCOSE!SODIUM”, “GOLIMUMAB”, “GOLODIRSEN”, “GONADORELIN”, “GOSERELIN”, “GRANISETRON”, “GUSELKUMAB”, “HALOPERIDOL”, “HEMIN”, “HEPARIN!SODIUM”, “HETASTARCH”, “HETASTARCH!SODIUM”, “HISTRELIN”, “HUMAN ALPHA 1 PROTEINASE INHIBITOR”, “HYALURONIC ACID”, “HYALURONIDASE”, “HYDRALAZINE”, “HYDROCORTISONE”, “HYDROMORPHONE”, “HYDROXYPROGESTERONE”, “HYDROXYZINE”, “HYLAN GEL POLYSACCHARIDE”, “HYOSCYAMINE”, “IBANDRONIC ACID”, “IBUPROFEN”, “IBUTILIDE”, “ICATIBANT”, “IDARUBICIN”, “IDURSULFASE”, “IFOSFAMIDE”, “IMIGLUCERASE”, “IMMUNOGLOBULIN ANTI-CLOSTRIDIUM TETANI TOXIN”, “IMMUNOGLOBULIN ANTI-CYTOMEGALOVIRUS”, “IMMUNOGLOBULIN ANTI-D (RHO)”, “IMMUNOGLOBULIN ANTI-HEPATITIS B”, “IMMUNOGLOBULIN ANTI-HEPATITIS B SURFACE ANTIGEN”, “IMMUNOGLOBULIN ANTI-THYMOCYTE”, “IMMUNOGLOBULIN ANTIVENOM CROTALIDAE SNAKES”, “IMMUNOGLOBULIN BASE”, “IMMUNOGLOBULIN BASE!VORHYALURONIDASE ALFA”, “INEBILIZUMAB”, “INFLIXIMAB”, “INOTUZUMAB OZOGAMICIN”, “INTERFERON ALFA-2B”, “INTERFERON BETA-1A”, “INTERFERON BETA-1B”, “INTERFERON GAMMA-1B”, “IPILIMUMAB”, “IRINOTECAN”, “IRON DEXTRAN”, “IRON FERRIC”, “ISATUXIMAB”, “ISAVUCONAZOLE”, “ITRACONAZOLE”, “IXABEPILONE”, “KETOROLAC”, “LANADELUMAB, “LANREOTIDE”, “LARONIDASE”, “LEFAMULIN”, “LEUPRORELIN”, “LEVETIRACETAM”, “LEVOCARNITINE”, “LEVOFLOXACIN”, “LEVONORGESTREL”, “LEVORPHANOL”, “LIDOCAINE”, “LINCOMYCIN”, “LINEZOLID”, “LONCASTUXIMAB TESIRINE”, “LONOCTOCOG ALFA”, “LORAZEPAM”, “LUMASIRAN”, “LURBINECTEDIN”, “LUSPATERCEPT”, “MAGNESIUM”, “MANNITOL”, “MARGETUXIMAB”, “MECASERMIN”, “MEDROXYPROGESTERONE”, “MELOXICAM”, “MELPHALAN”, “MELPHALAN FLUFENAMIDE”, “MEPIVACAINE”,“MEPOLIZUMAB”, “MEROPENEM”, “MEROPENEM!VABORBACTAM”, “MESNA”, “METHADONE”, “METHOCARBAMOL”, “METHOTREXATE”, “METHOXY POLYETHYLENE GLYCOL-EPOETIN BETA”, “METHYLERGOMETRINE”, “METHYLNALTREXONE BROMIDE”, “METHYLPREDNISOLONE”, “METOCLOPRAMIDE”, “MICAFUNGIN”, “MIDAZOLAM”, “MILRINONE”, “MINOCYCLINE”, “MITOMYCIN”, “MITOXANTRONE”, “MOGAMULIZUMA”, “MOMETASON0E”, “MOMETASONE”, “MONOETHANOLAMINE OLEATE”, “MOROCTOCOG ALFA”, “MORPHINE”, “MOXETUMOMAB PASUDOTOX”, “MOXIFLOXACIN”, “NALBUPHINE”, “NALOXONE”, “NALTREXONE”, “NANDROLONE”, “NATALIZUMAB”, “NAXITAMAB”, “NECITUMUMAB”, “NELARABINE”, “NEOSTIGMINE”, “NESIRITIDE”, “NIVOLUMAB”, “NUSINERSEN”, “OBINUTUZUMAB”, “OCRELIZUMAB”, “OCRIPLASMIN”, “OCTREOTIDE”, “OFATUMUMAB”, “OLANZAPINE”, “OLARATUMAB”, “OMACETAXINE MEPESUCCINATE”, “OMADACYCLINE”, “OMALIZUMAB”, “ONASEMNOGENE ABEPARVOVEC”, “ONDANSETRON”, “OPRELVEKIN”, “ORITAVANCIN”, “ORPHENADRINE”, “OXACILLIN”, “OXALIPLATIN”, “OXYMORPHONE”, “OXYTOCIN”, “PACLITAXEL”, “PALIFERMIN”, “PALIPERIDONE”, “PALONOSETRON”, “PAMIDRONIC ACID”, “PANITUMUMAB”, “PAPVERINE”, “PARACETAMOL”, “PARICALCITOL”, “PASIREOTIDE”, “PATISIRAN”, “PEGADEMASE”, “PEGAPTANIB”, “PEGASPARGASE”, “PEGFILGRASTIM”, “PEGINTERFERON ALFA-2A”, “PEGINTERFERON ALFA-2A!RIBAVIRIN”, “PEGLOTICASE”, “PEMBROLIZUMAB”, “PEMETREXED”, “PENICILLIN G”, “PENTAZOCINE”, “PENTOBARBITAL”, “PENTOSTATIN”, “PERAMIVIR”, “PERPHENAZINE”, “PERTUZUMAB”, “PERTUZUMAB!TRASTUZUMAB!VORHYALURONIDASE ALFA”, “PETHIDINE”, “PETHIDINE!PROMETHAZINE”, “PHENOBARBITAL”, “PHENTOLAINE”, “PHENYLEPHRINE”, “PHENYTOIN”, “PHYTOMENADIONE”, “PIPERACILLIN!TAZOBACTAM”, “PLAZOMICIN”, “PLERIXAFOR”, “POLATUZUMAB

© 2024 Callaway Kim K et al. *JAMA Network Open.*

	VEDOTIN", "PORFIMER SODIUM", "POTASSIUM", "PRALATREXATE", "PRALIDOXIME", "PREDNISOLONE", "PROCAINAMIDE", "PROCHLORPERAZINE", "PROGESTERONE", "PROMETHAZINE", "PROPOFOL", "PROPRANOLOL", "PROTAMINE", "PROTEIN C", "PROTIRELIN", "PYRIDOXINE", "RAMUCIRUMAB", "RANIBIZUMAB", "RANITIDINE", "RASBURICASE", "RAVULIZUMAB", "REGADENOSON", "REMDESIVIR", "RESLIZUMAB", "RETEPLASE", "RILONACEPT", "RISPERIDONE", "RITUXIMAB", "RITUXIMAB!VORHYALURONIDASE ALFA", "ROLAPITANT", "ROMIDEPSIN", "ROMIPLOSTIM", "ROMOSOZUMAB", "ROPIVACAINE", "SACITUZUMAB GOVITECAN", "SALBUTAMOL", "SARGRAMOSTIM", "SEBELIPASE ALFA", "SECRETIN", "SILTUXIMAB", "SIMOCTOCOG ALFA", "SINCALIDE", "SIROLIMUS", "SOMATROPIN", "STREPTOMYCIN", "STREPTOZOCIN", "SUMATRIPTAN", "SUXAMETHONIUM", "TACROLIMUS", "TAFASITAMAB", "TAGRAXOFUSP", "TALIGLUCERASE ALFA", "TALIMOGENE LAHERPAREPVEC", "TEDIZOLID", "TELAVANCIN", "TEMOZOLOMIDE", "TEMSIROLIMUS", "TENECTEPLASE", "TEPROTUMUMAB", "TERBUTALINE", "TERIPARATIDE", "TESTOSTERONE", "TETRACOSACTIDE", "TETRACYCLINE", "THEOPHYLLINE", "THIAMINE", "THIOTEPA" , "THYROTROPIN", "TIGECYCLINE", "TILDRAKIZUMAB", "TIROFIBAN", "TISOTUMAB", "TOBRAMYCIN", "TOCILIZUMAB", "TOPOTECAN", "TORASEMIDE", "TRABECTEDIN", "TRASTUZUMAB", "TRASTUZUMAB DERUXTECAN", "TRASTUZUMAB EMTANSINE", "TREPROSTINIL" , "TRIAMCINOLONE", "TRIAMCINOLONE ACETONIDE", "TRIAMCINOLONE HEXACETONIDE", "TRIFLUOPERAZINE", "TRILACICLIB", "TRIMETHOBENZAMIDE", "TRIPTORELIN", "TUROCTOCOG ALFA", "TUROCTOCOG ALFA PEGOL", "UREA", "UROFOLLITROPIN", "USTEKINUMAB", "VACCINE, BACILLUS CALMETTE-GUERIN", "VALRUBICIN", "VANCOMYCIN", "VEDOLIZUMAB", "VELAGLUCERASE ALFA", "VERTEPORFIN", "VESTRONIDASE ALFA", "VILTOLARSEN", "VINBLASTINE", "VINCRISTINE", "VINORELBINE", "VONICOG ALFA", "VORETIGENE NEPARVOVEC", "VORICONAZOLE", "ZICONOTIDE", "ZIDOVUDINE", "ZIPRASIDONE", "ZOLEDRONIC ACID", "DACARBAZINE", "EPTACOG ALFA (ACTIVATED) ", "ETONOGESTREL", "TOBRAMYCIN"
Marketing category and years since market entry	Marketing category and age category were manually assigned using FDA's National Drug Code (NDC) directory downloaded in August 2022. Drugs were characterized as: (a) brand-name (all NDCs under New Drug Application license), (b) generic prior to January 2017 (first Abbreviated New Drug Application approved on or before January 2017), (c) generic approved during study period (first Abbreviated New Drug Application approved between January 2017 and December 2021), or (d) vaccines/biologics (Biologic License Application). Drugs not found in the NDC directory were assigned to missing.
Annual sales volume	Assigned using the sum of ex-manufacturer sales in the 6 months prior to each drug's index date. Ex-manufacturer sales represent the price level from manufacturer to wholesaler (ex-factory level, ex-manufacturer level, wholesaler purchase level).
Type of supply chain issue	Assigned based on the source of data for each supply chain issue report:<br>• "Shortage-probable" reports were those downloaded either from the FDA Drug Shortage website (https://www.accessdata.fda.gov/scripts/drugshortages/default.cfm), CBER's shortage website (https://www.fda.gov/vaccines-blood-biologics/safety-availability-biologics/cber-regulated-products-current-shortages) or from ASHP (https://www.ashp.org/drug-shortages/current-shortages?loginreturnUrl=SSOCheckOnly).<br>• Recalls were downloaded from FDA's Market Withdrawals and Safety Alerts website (https://www.fda.gov/safety/recalls-market-withdrawals-safety-alerts) or from CBER's recall website (https://www.fda.gov/vaccines-blood-biologics/safety-availability-biologics/recalls-biologics).<br>• Discontinuations were those reports on FDA's Drug Shortage website where the Status variable was "To Be Discontinued".

© 2024 Callaway Kim K et al. *JAMA Network Open.*

Supply chain issue index date	For FDA reports, the supply chain issue index date was the initial posting date to FDA's website. ASHP index dates were assigned using the creation date at the end of each report.
Supply chain issue end date	For FDA and ASHP shortage-probable reports, the end date of each supply chain issue was defined using the posting date on the given organization's website on which the status of that report changed from "Current" to "Resolved". Reports were downloaded daily to ensure full capture of these changes. Discontinuations and recalls did not have a reported "resolved" status on FDA's website. Because we expected discontinuations and recalls to have an immediate impact on medication supplies, we therefore assigned each of these reports a duration equal to 90 days.
Reason for supply chain issue	For FDA shortage-probable reports, the issue reason was assigned using the "Reason for Shortage" variable on FDA's website. Reports were assigned to the "Missing or Unspecified" category if this variable was left blank or had an unspecified reason.<br><br>Because ASHP reasons are not standardized, the first and senior author manually reviewed all ASHP reports and assigned these to a single reason category which was consistent with FDA's list:<br>• Any ASHP report which mentioned product mix-ups, mislabeling, potency concerns, manufacturing delays, or other manufacturing changes was assigned to the "Manufacturing, packaging, or shipping" category<br>• Any ASHP report which mentioned toxic ingredients such as nitrosamines was assigned to the "Impurities" category<br>• Any ASHP report which mentioned microbial contamination or other sterility concerns was assigned to "Lack of sterility"<br>• Any ASHP report which mentioned a drug discontinuation was assigned to "Discontinuation" as the reason for the issue (Note: ASHP reports were still considered as shortage-probable reports vs. discontinuations only)<br>• All other ASHP reports were assigned to the "Other" category which included ingredient shortages, natural disasters, increased demand, and other rare events.<br><br>For ASHP reports where different companies reported different reasons, we assigned the following priority based on the likely temporal sequence/severity of these events: API shortage > manufacturing delay > other event such as increased demand or discontinuation.

© 2024 Callaway Kim K et al. *JAMA Network Open.*

e-Table 3: Characteristics of Supply Chain Issue Reports, by COVID-19 Pandemic Period

e-Table 3 presents descriptive statistics on supply chain issue reports, by COVID-19 pandemic period. Reports were grouped based on their issuance date as occurring pre-pandemic (January 2017 – January 2020), immediately after the WHO public health emergency announcement (February 2020 – April 2020) and during the first pandemic year (May 2020 – September 2021). Generally, characteristics of exposed drugs with supply chain issue reports were similar across the three pandemic periods; however, we observed an increase in the proportion of issues that involved discontinuations over time, as well as an increase in issues with no/unspecified reason. The average change in purchased units post-report was largest for reports issued from February to April 2020.

Variable	Supply Chain Issue Reports (No. = 731)		
	Jan. 2017 – Jan. 2020 (No. = 489)	Feb. 2020 – Apr. 2020 (No. = 57)	May 2020 – Sept. 2021 (No. = 185)
Reporting agency, no. (%)			
FDA	379 (78)	54 (95)	155 (84)
ASHP	235 (48)	22 (39)	70 (38)
Type of Issue, no. (%)			
Shortage-probable	261 (53)	25 (44)	77 (42)
Recall	104 (21)	13 (23)	14 (8)
Discontinuation	221 (45)	33 (58)	113 (61)
Median (IQR) supply chain issue duration in months[b]	4.5 (3,21)	3 (3,23.9)	3 (3,11.8)
Duration category, no. (%)			
<6 months	253 (52)	31 (54)	117 (63)
6-11 months	59 (12)	4 (7)	22 (12)
12-23 months	68 (14)	8 (14)	33 (18)
24-35 months	30 (6)	14 (25)	13 (7)
≥36 months	79 (16)	0 (0)	0 (0)
Reason for supply chain issue, no. (%)			
No or unspecified reason	153 (31)	24 (42)	87 (47)
Manufacturing, packaging, or shipping issues	112 (23)	13 (23)	38 (21)
Business Decision	82 (17)	2 (4)	24 (13)
Discontinuation of the manufacture of the drug	57 (12)	5 (9)	11 (6)
Impurities or lack of sterility	45 (9)	4 (7)	6 (3)
Other Events[a]	40 (8)	9 (16)	19 (10)
Formulation, no. (%)			
Oral	268 (55)	34 (60)	101 (55)
Parenteral	196 (40)	18 (32)	72 (39)
Ophthalmic or otic	17 (3)	1 (2)	9 (5)
Inhaled	8 (2)	4 (7)	3 (2)
≥ 5 manufacturers at baseline, no. (%)	247 (51)	37 (65)	94 (51)
WHO essential medicine, no. (%)	204 (42)	33 (58)	79 (43)
Provider-administered drug, no. (%)	132 (27)	12 (21)	53 (29)
Marketing Category, no. (%)			
Generic available	416 (85)	51 (89)	149 (81)
Brand-name only	37 (8)	5 (9)	23 (12)
Other or unknown	35 (7)	1 (2)	12 (6)
Baseline sales <5 million USD[j], no. (%)	150 (31)	13 (23)	71 (38)
Median (IQR) drug age	19.5 (12.8,29.5)	23 (14.9,31.7)	22.4 (14.9,33.6)
Age category, %			
<5 years	34 (7)	1 (2)	5 (3)
5-9 years	49 (10)	7 (12)	22 (12)

© 2024 Callaway Kim K et al. *JAMA Network Open.*

10-19 years	167 (34)	19 (33)	52 (28)
≥20 years	239 (49)	30 (53)	106 (57)
Mean (95% CI) change in MIDAS units, relative to 3 months pre-report-issuance			
Quarter comprising 1-3 months post-report	-6.3% (-16.0, 3.4%)	-13.1% (-21.8, -4.4%)	-7.1% (-22.6, 8.3%)
Quarter comprising 4-6 months post-report	-4.3% (-7.9, -0.01%)	-14.9% (-21.6, -8.3%)	-4.4% (-11.3, 2.4%)

Abbreviations: No. = number; IQR = interquartile range

a. Among other events, increased demand for the drug was the most commonly listed reason, accounting for 24 out of 40 reports pre-pandemic (60%), 3 of 9 from February 2020 to April 2020 (30%), and 9 of 19 post-May 2020 (47%).

© 2024 Callaway Kim K et al. *JAMA Network Open.*

e-Table 4: Characteristics of Supply Chain Issue Reports, by Report Type[a]

e-Table 4 presents descriptive statistics for exposed drugs with supply chain issue reports, by report type. We defined supply chain issue reporting episodes using all available public databases (see e-Figure 2). Among episodes with reports from multiple databases, we prioritized FDA shortage-probable and ASHP shortage-probable reports for this table. Compared to recall-only and discontinuation-only episodes, shortage-probable episodes were more likely to involve parenteral drugs, and less likely to have ≥5 manufacturers in the 6 months pre-report-issuance. Manufacturing issues comprised most recalls-only report episodes, and business decisions comprised most discontinuation-only episodes.

Variable	Supply Chain Issue Reports (No. = 731)		
	**Any Shortage-probable Report (No. = 363)**	**Recall Report Only (No. = 91)**	**Discontinuation Report Only (No. = 277)**
Time period, no. (%)			
January 2017 – January 2020	261 (72)	69 (76)	159 (57)
February 2020 – April 2020	25 (7)	9 (10)	23 (8)
May 2020 – September 2021	77 (21)	13 (14)	95 (34)
Reporting agency, no. (%)			
FDA	220 (61)	91 (100)	277 (100)
ASHP	327 (90)	0 (0)	0 (0)
Type of Issue, no. (%)			
Shortage-probable	363 (100)	0 (0)	0 (0)
Recall	40 (11)	91 (100)	0 (0)
Discontinuation	84 (23)	6 (7)	277 (100)
Median (IQR) supply chain issue duration in months[b]	17.9 (8.4,33.4)	3 (3,3)	3 (3,3)
Duration category, no. (%)			
<6 months	62 (17)	80 (88)	259 (94)
6-11 months	56 (15)	11 (12)	18 (6)
12-23 months	109 (30)	0 (0)	0 (0)
24-35 months	57 (16)	0 (0)	0 (0)
≥36 months	79 (22)	0 (0)	0 (0)
Reason for supply chain issue, no. (%)			
No or unspecified reason	91 (25)	0 (0)	173 (62)
Manufacturing, packaging, or shipping issues	119 (33)	44 (48)	0 (0)
Business Decision	4 (1)	0 (0)	104 (38)
Discontinuation of the manufacture of the drug	73 (20)	0 (0)	0 (0)
Impurities or lack of sterility	13 (4)	42 (46)	0 (0)
Other Events	63 (17)	5 (5)	0 (0)
Formulation, no. (%)			
Oral	137 (38)	50 (55)	216 (78)
Parenteral	201 (55)	38 (42)	47 (17)
Ophthalmic or otic	16 (4)	2 (2)	9 (3)
Inhaled	9 (2)	1 (1)	5 (2)
≥ 5 manufacturers at baseline, no. (%)	138 (38)	53 (58)	187 (68)
WHO essential medicine, no. (%)	154 (42)	43 (47)	119 (43)
Provider-administered drug, no. (%)	138 (38)	29 (32)	30 (11)
Marketing Category, no. (%)			
Generic available	294 (81)	77 (85)	245 (88)
Brand-name only	37 (10)	6 (7)	22 (8)
Other or unknown	31 (9)	8 (9)	9 (3)

© 2024 Callaway Kim K et al. *JAMA Network Open.*

Baseline sales <5 million USD [j], no. (%)	133 (37)	15 (16)	86 (31)
Median (IQR) drug age	21.8 (14.3,32.2)	16.7 (10.6,31)	19.6 (14.8,29.3)
Age category, %			
<5 years	19 (5)	7 (8)	14 (5)
5-9 years	34 (9)	14 (15)	30 (11)
10-19 years	110 (30)	29 (32)	99 (36)
≥20 years	200 (55)	41 (45)	134 (48)
Mean (95% CI) change in MIDAS units, relative to 3 months pre-report-issuance			
Quarter comprising 1-3 months post-report	-3.5% (-9.2, 2.0%)	0.001% (-4.3, 4.6%)	-8.9% (-11.9, -5.8%)
Quarter comprising 4-6 months post-report	-7.0% (-15.6, 1.3%)	3.5% (-26.0, 32.9%)	-11.4% (-22.6, -0.001%)

Abbreviations: No. = number; IQR = interquartile range (a) Because many issues contained multiple reports, we assigned the following priority: any shortage > any recall > any discontinuation. (b) Because FDA does not consistently report end dates for recalls and discontinuations, these were assigned a duration equal to 3 mons.

© 2024 Callaway Kim K et al. *JAMA Network Open.*

e-Table 5: Characteristics of Supply Chain Issue Reports and Matched Comparison Drugs, by MIDAS Supply Disruption Status

e-Table 5 presents descriptive statistics for exposed drugs with supply chain issue reports, by supply disruption status in MIDAS. Drugs were considered to be in shortage if the percentage decrease in units met the 33% (meaningful) or 66% (severe) cut-off in either Q1 (months 1-3) or Q2 (months 4-6), relative to the quarter pre-report-issuance; some drugs on shortage in Q1 may therefore have gone off shortage in Q2.

Variable	Supply Chain Issue Reports (No. = 731)			Matched Comparison Drugs (No. = 7290)		
	No Shortage (No. = 618)	Meaningful Shortage ≥33% (No. = 113)	Severe Shortage ≥66% (No. = 63)	No Shortage (No. = 6803)	Meaningful Shortage ≥33% (No. = 491)	Severe Shortage ≥66% (No. = 188)
Mean (95% CI) change in MIDAS units, relative to 3 months pre-report-issuance						
Quarter comprising 1-3 months post-report	5.3% (2.7, 8.0%)	-60.9% (-67.1, -54.6%)	-81.3% (-87.6, -75.0%)	880% (-747, 252%)	-46.2% (-52.2, -40.3%)	-60.6% (-75.7, -45.6%)
Quarter comprising 4-6 months post-report	7.8% (-0.01, 16.4%)	-55.4% (-66.5, -44.2%)	-70.9% (-89.1, -52.66%)	46.2% (14.1, 78.2%)	-14.3% (-77.6, 49.0%)[d]	19.7% (-150.5, 190%)[d]
Time period, no. (%)						
Jan. 2017 – Jan. 2020	415 (67)	74 (65)	41 (65)			
Feb. 2020 – April 2020	45 (7)	12 (11)	3 (5)			
May 2020 – Sept. 2021	158 (26)	27 (24)	19 (30)			
Reporting agency[a], no. (%)						
FDA	493 (80)	95 (84)	58 (92)			
ASHP	257 (42)	70 (62)	31 (49)			
Type of issue[a], no. (%)						
Shortage	280 (45)	83 (73)	43 (68)			
Recall	118 (19)	13 (12)	4 (6)			
Discontinuation	319 (52)	48 (42)	33 (52)			
Median (IQR) supply chain issue duration in months	3 (3,16.5)	13.8 (3,23.4)				
Duration category, no. (%)						
<6 months	365 (59)	36 (32)	21 (33)			
6-11 months	67 (11)	18 (16)	9 (14)			
12-23 months	76 (12)	33 (29)	19 (30)			
24-35 months	47 (8)	10 (9)	7 (11)			
≥36 months	63 (10)	16 (14)	7 (11)			
Reason for supply chain issue[b], no. (%)						
No or unspecified reason	225 (36)	39 (35)	21 (33)			

© 2024 Callaway Kim K et al. *JAMA Network Open.*

Manufacturing, packaging, or shipping issues	134 (22)	29 (26)	17 (27)			
Business Decision	100 (16)	8 (7)	8 (13)			
Discontinuation of the manufacture of the drug	60 (10)	13 (12)	5 (8)			
Impurities or lack of sterility	47 (8)	8 (7)	3 (5)			
Other Events	52 (8)	16 (14)	9 (14)			
Formulation, no. (%)						
Oral	349 (56)	54 (48)	35 (56)	3790 (56)	211 (43)	76 (41)
Parenteral	238 (39)	48 (42)	22 (35)	2422 (36)	230 (47)	96 (51)
Ophthalmic or otic	19 (3)	8 (7)	4 (6)	408 (6)	38 (8)	10 (5)
Inhaled	12 (2)	3 (3)	2 (3)	185 (3)	12 (2)	6 (3)
≥ 5 manufacturers at baseline[c], no. (%)	365 (59)	13 (12)	4 (6)	2180 (32)	45 (9)	7 (4)
WHO essential medicine, no. (%)	287 (46)	29 (26)	10 (16)	2070 (30)	129 (26)	43 (23)
Provider-administered drug, no. (%)	169 (27)	28 (25)	14 (22)	1624 (24)	106 (22)	41 (22)
Marketing Category, no. (%)						
Generic available	545 (88)	71 (63)	36 (57)	4124 (61)	273 (56)	99 (54)
Brand-name only	34 (6)	31 (27)	21 (33)	1674 (25)	138 (28)	57 (31)
Other or unknown	37 (6)	11 (10)	6 (10)	968 (14)	78 (16)	32 (17)
Baseline sales <5 million USD[c], no. (%)	154 (25)	80 (71)	48 (76)	1857 (27)	301 (61)	142 (76)
Median (IQR) drug age in years	21.1 (14.9,30.8)	17 (9,28.5)	14.8 (7.5,28.6)	14.5 (7.8,23.4)	13.85 (6.8,25.3)	12.05 (5.9,24.6)
Age category, %						
<5 years	25 (4)	15 (13)	12 (19)	899 (13)	77 (16)	34 (18)
5-9 years	60 (10)	18 (16)	13 (21)	1369 (20)	99 (20)	43 (23)
10-19 years	206 (33)	32 (28)	15 (24)	2267 (33)	145 (30)	50 (27)
≥20 years	327 (53)	48 (42)	23 (37)	2268 (33)	166 (34)	58 (31)

Abbreviations: No. = number; IQR = interquartile range

a. Categories are not mutually exclusive since a single drug supply issue episode could include multiple events.

b. Other events included regulatory delays, shortages of active or inactive ingredients, demand increases, or other miscellaneous reasons.

c. Measured in MIDAS for the 6 months immediately prior to supply issue event initiation (baseline period).

d. Drugs were considered to be in shortage if the percentage decrease in units met the 33% (meaningful) or 66% (severe) cut-off in *either* Q1 (months 1-3) or Q2 (months 4-6), relative to the quarter pre-report-issuance; some drugs on shortage in Q1 may therefore have gone off shortage in Q2.

© 2024 Callaway Kim K et al. *JAMA Network Open.*

e-Figure 4: Unadjusted Probabilities of Meaningful (≥33%) and Severe (≥66%) Shortages, by Report Type

e-Figure 4 presents the unadjusted percentage of supply chain issue reports which were associated with meaningful (≥33%) and severe (≥66%) drug shortages within 6 months, by report characteristics.

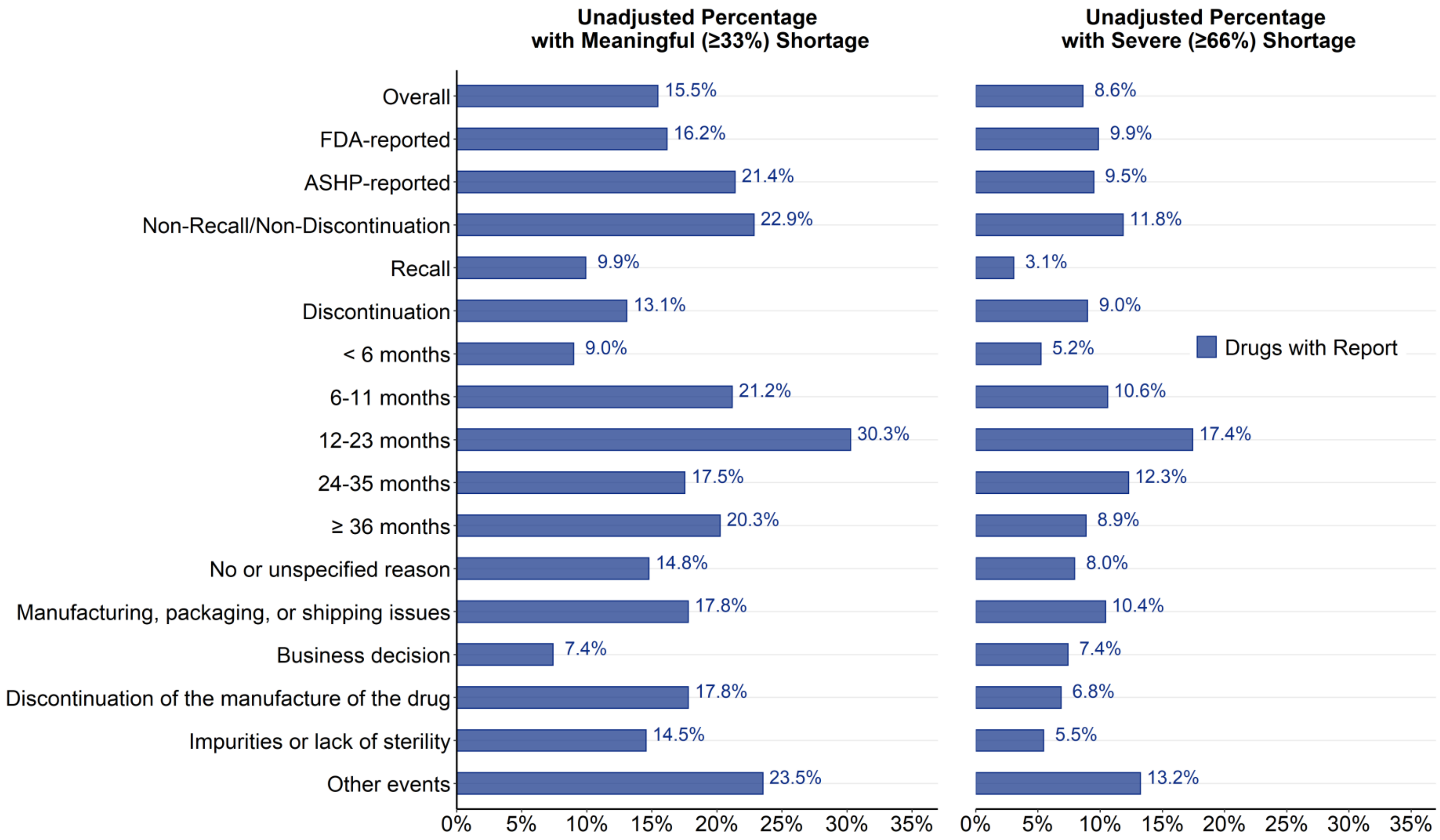

© 2024 Callaway Kim K et al. *JAMA Network Open.*

e-Figure 5: Unadjusted Proportion of Incident Supply Chain Issue Reports and Matched Comparison Drugs Associated with Severe (≥66%) Drug Shortages within 6 months[a], by Quarter, 2017-2021

e-Figure 5 presents the unadjusted proportion of incidence supply chain issue reports and matched comparison drugs which were associated with drug shortages within 6 months. Reports were grouped according to their issuance date. The grey bars show the number of incident reports per calendar quarter (primary y-axis). The blue and yellow lines on the secondary y-axis are the proportion of incident reports (blue) and matched unexposed comparison drugs (yellow) which were subsequently associated with a severe (≥66%) drug shortage within 6 months.

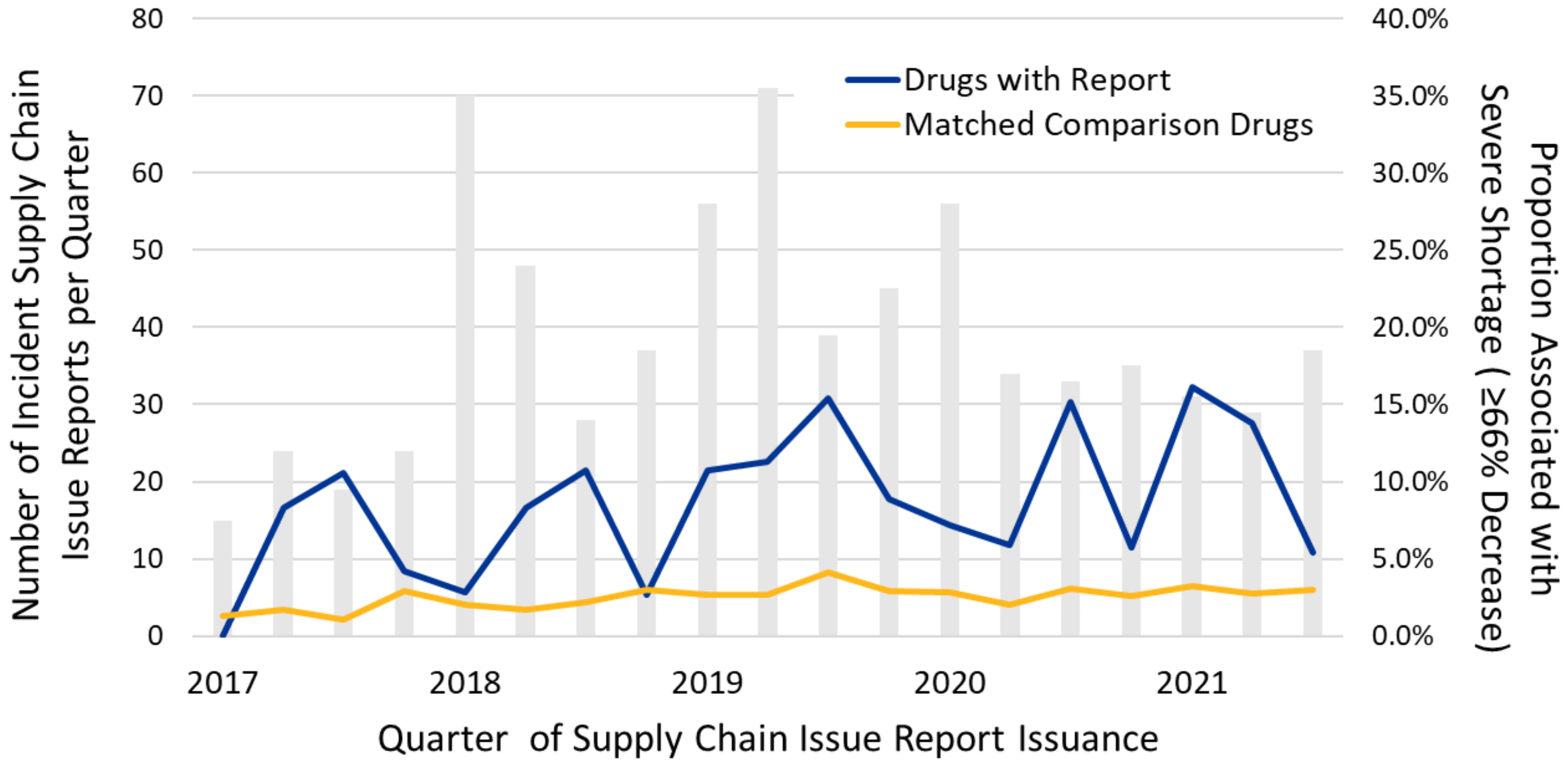

a. Severe supply disruption defined as a ≥66% decrease in total standardized units within two quarters post-supply-issue-initiation, relative to three months pre-supply-issue.

© 2024 Callaway Kim K et al. *JAMA Network Open.*

e-Figure 6: Marginal Odds Ratios and Predicted Probabilities of Severe (≥66%) Shortages within 6 months, Supply Chain Issue Reports vs. Matched Comparison Drugs, Pre- vs. During-COVID-19 Pandemic[a,b]

e-Figure 6 presents the post-hoc marginal odds ratios and marginal predicted probabilities obtained from fitting a random effect logistic regression model for our secondary outcome of severe (≥66%) shortages within 6 months of an incident supply chain issue report. A marginal odds ratio of 6.5 indicates that, on average, drugs with supply chain issue reports had 6.5 times the odds of incident severe shortages as drugs without supply chain issue reports. Predicted probabilities represent absolute incidence. Estimated marginal effects were obtained by averaging over drug formulation, years since FDA approval, marketing category, WHO essential medicine status, provider vs. self-administration, total sales, and number of manufacturers at baseline.

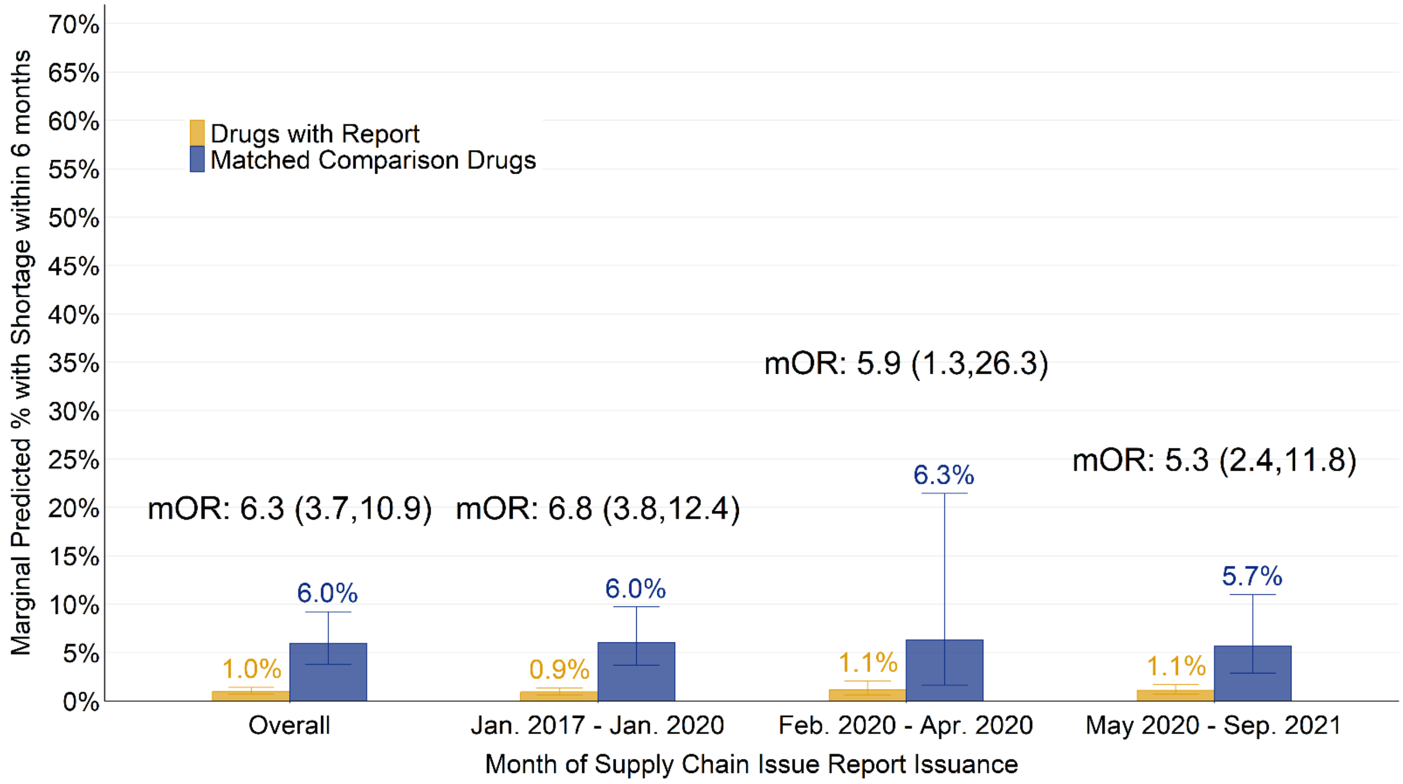

Abbreviations: mOR = marginal odds ratio

a. Reports were grouped according to their issuance date.
b. Severe shortages defined as a ≥66% decrease in total standardized units within two quarters post-supply-issue-initiation, relative to three months pre-supply-issue.

© 2024 Callaway Kim K et al. *JAMA Network Open.*

e-Figure 7: Marginal Odds Ratios and Predicted Probabilities of Severe (≥66%) Shortages within 6 months, Supply Chain Issue Reports vs. Matched Comparison Drugs, by Drug Characteristics[a]

e-Figure 7 presents the post-hoc marginal odds ratios and marginal predicted probabilities obtained from fitting a random effect logistic regression model for our secondary outcome of severe (≥66%) shortages within 6 months of an incident supply chain issue report, including interaction terms by drug and supply chain characteristics. The "Overall" marginal odds ratio of 6.3 indicates that, on average, drugs with supply chain issue reports had 6.3 times the odds of experiencing severe shortages within 6 months as drugs without supply chain issue reports. All other marginal odds ratios depict the relative odds of experiencing severe shortages among drugs with a given characteristic: for example, oral drugs with supply chain issues had on average 12.7 times the odds of experiencing severe shortages as oral drugs without supply chain issues. Predicted probabilities represent absolute incidences. Estimated marginal effects were obtained by averaging over drug formulation, years since FDA approval, marketing category, WHO essential medicine status, provider vs. self-administration, total sales, and number of manufacturers at baseline.

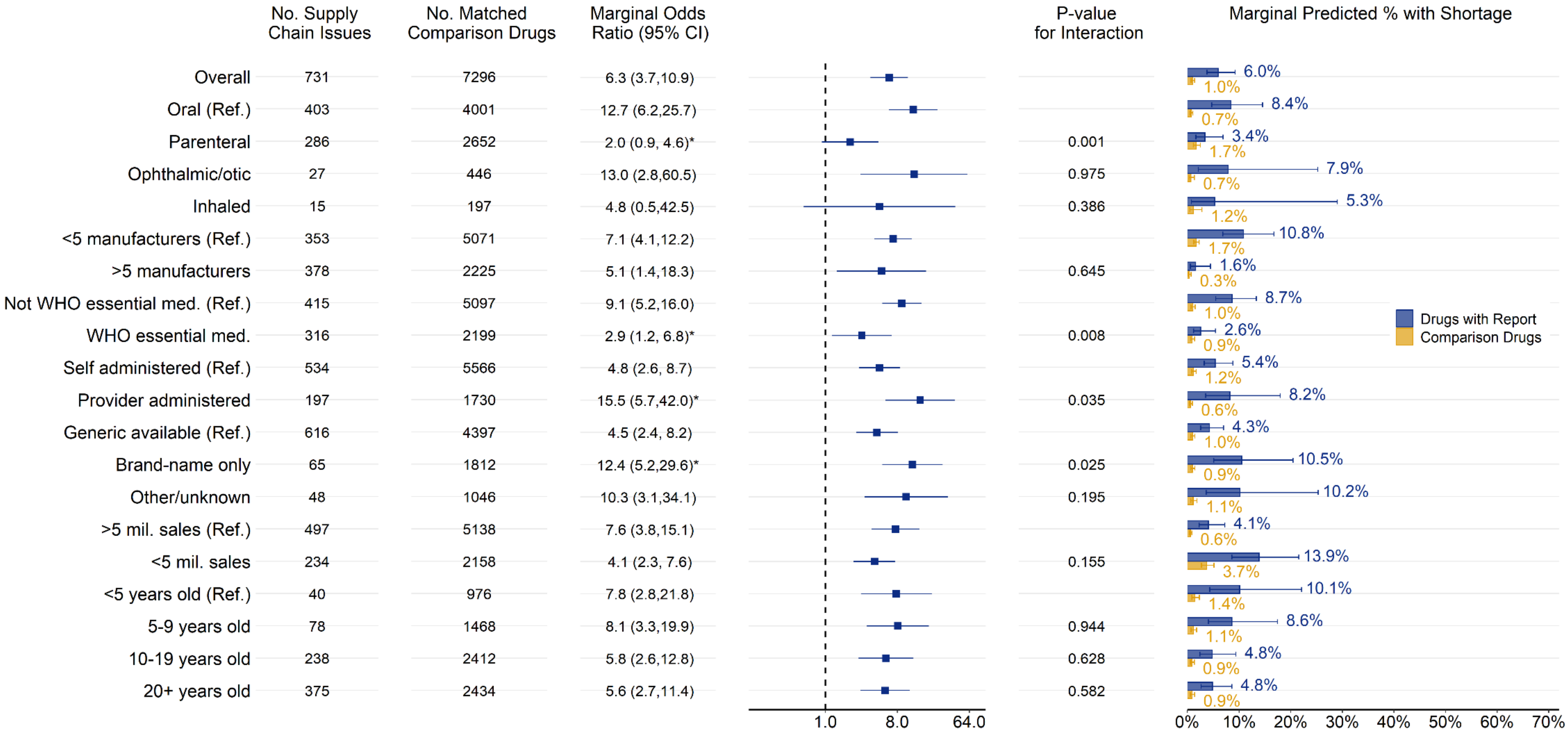

© 2024 Callaway Kim K et al. *JAMA Network Open.*

Abbreviations: Ref. = reference category; WHO = World Health Organization * **denotes significant interaction effect**

© 2024 Callaway Kim K et al. *JAMA Network Open.*

e-Table 6: Random Effects Logistic Regression Estimates, Odds of Meaningful (≥33%) and Severe (≥66%) Shortages, Supply Chain Issue Reports vs. Matched Comparison Drugs

e-Table 6 presents the full regression output for our random effects logistic regression models looking at our primary outcome of meaningful (≥33%) and secondary outcome of severe (≥66%) shortages within 6 months after an incident supply chain issue report. Odds ratios reflect the relationship between having the characteristic of interest and the outcome, conditional on other covariates and set-specific random effects. Due to a mathematical property of odds ratios called non-collapsibility, the conditional odds ratios in this table will be larger than the reported marginal odds ratios in Figure 3 and Figure 4, which are effects estimated by "collapsing" or averaging over all other covariates and set-specific random effects.

	**Meaningful Shortages**		**Severe Shortages**	
**Variable**	**Odds Ratio (95% CI)**	**p-value**	**Odds Ratio (95% CI)**	**p-value**
Supply Issue Report versus matched comparison drugs	4.53 (1.61,12.74)	0.004	17.01 (5.02,57.6)	<0.001
Time period				
January 2017 – January 2020	Ref.	Ref.	Ref.	Ref.
February 2020 – April 2020	2.55 (1.85,3.5)	<0.001	1.23 (0.7,2.16)	0.47
May 2020 – September 2021	0.9 (0.7,1.16)	0.42	1.21 (0.85,1.73)	0.30
Supply Issue * Time Period				
January 2017 – January 2020	Ref.	Ref.	Ref.	Ref.
February 2020 – April 2020	1.28 (0.51,3.22)	0.60	0.86 (0.18,4)	0.85
May 2020 – September 2021	0.75 (0.4,1.42)	0.38	0.78 (0.36,1.7)	0.54
Formulation				
Oral	Ref.	Ref.	Ref.	Ref.
Parenteral	2.26 (1.72,2.97)	<0.001	2.37 (1.59,3.53)	<0.001
Ophthalmic or otic	1.37 (0.94,2.02)	0.11	0.91 (0.46,1.82)	0.79
Inhaled	1.22 (0.65,2.3)	0.54	1.61 (0.66,3.92)	0.30
Supply Issue * Formulation				
Oral	Ref.	Ref.	Ref.	Ref.
Parenteral	**0.35 (0.16,0.76)**	**0.008**	**0.16 (0.06,0.46)**	**0.001**
Ophthalmic or otic	1.52 (0.46,5.01)	0.49	1.02 (0.22,4.69)	0.975
Inhaled	0.46 (0.07,3.04)	0.42	0.38 (0.04,3.4)	0.39
≥ 5 manufacturers at baseline	0.34 (0.24,0.48)	<0.001	0.19 (0.09,0.41)	<0.001
Supply Issue * ≥ 5 manufacturers	0.59 (0.26,1.35)	0.21	0.72 (0.17,2.96)	0.65
WHO essential medicine	0.98 (0.78,1.22)	0.83	0.88 (0.61,1.27)	0.50
Supply Issue* WHO essential med.	**0.49 (0.27,0.88)**	**0.02**	**0.32 (0.13,0.74)**	**0.008**
Provider-administered drug	0.48 (0.36,0.64)	<0.001	0.49 (0.32,0.75)	0.001
Supply Issue * Provider-administered drug	2.14 (0.96,4.74)	0.06	**3.23 (1.09,9.62)**	**0.04**
Marketing Category				
Generic (ANDA approval)	Ref.	Ref.	Ref.	Ref.
Brand-name (NDA approval)	0.92 (0.71,1.2)	0.55	0.95 (0.65,1.4)	0.80
Other or unknown	0.92 (0.66,1.29)	0.64	1.1 (0.66,1.83)	0.71
Supply Issue * Marketing Category				
Generic (ANDA approval)	Ref.	Ref.	Ref.	Ref.
Brand-name (NDA approval)	**3.32 (1.54,7.15)**	**0.002**	**2.78 (1.14,6.81)**	**0.03**
Other or unknown	2.6 (0.99,6.8)	0.05	2.31 (0.65,8.17)	0.20
Baseline sales <5 million USD	3.65 (2.95,4.52)	<0.001	6.92 (4.79,9.99)	<0.001

© 2024 Callaway Kim K et al. *JAMA Network Open.*

Supply Issue * Baseline sales <5 million USD	1.14 (0.62,2.08)	0.68	0.54 (0.24,1.26)	0.15
Age Category				
<5 years	Ref.	Ref.	Ref.	Ref.
5-9 years	0.86 (0.62,1.19)	0.37	0.8 (0.5,1.29)	0.36
10-19 years	0.82 (0.59,1.13)	0.23	0.61 (0.37,0.99)	0.05
≥20 years	0.85 (0.61,1.19)	0.35	0.63 (0.38,1.03)	0.07
Supply Issue * Age Category				
< 5 years	Ref.	Ref.	Ref.	Ref.
5-9 years	1.13 (0.39,3.34)	0.82	1.04 (0.31,3.51)	0.94
10-19 years	0.96 (0.34,2.66)	0.93	0.74 (0.22,2.48)	0.63
≥20 years	0.93 (0.34,2.54)	0.89	0.72 (0.22,2.32)	0.58
Intercept	0.04 (0.03,0.06)	<0.001	0.01 (0.01,0.02)	<0.001
Distributions of Set-Specific Random Effects				
ICC (SE)	0.07 (0.03)		0.06 (0.05)	

Abbreviations: SE = standard error; ICC = intraclass correlation coefficient; CI = confidence interval**, bold denotes significant interaction effect**

© 2024 Callaway Kim K et al. *JAMA Network Open.*

e-Figure 8: Marginal Odds Ratios and Predicted Probabilities of Meaningful (≥33%) and Severe (≥66%) Shortages for Supply Chain Issues vs. Matched Comparison Drugs, Sensitivity Analyses Using Different Outcome Definitions and Inclusion Criteria

e-Figure 8 presents the post-hoc marginal odds ratios and marginal predicted probabilities obtained from our sensitivity analyses. Within each panel, the first row repeats the predicted marginal odds ratios and probabilities for meaningful (≥33%, Panel A) and severe (≥66%, Panel B) shortages from our main models. The second row represents our first sensitivity analysis which extended our post-report period from 2 quarters (6 months) to 3 quarters (9 months). We did not observe any substantial changes for meaningful or severe shortages using this longer time. The third row is the results from a model without interaction terms; because our subsequent sensitivity analysis had smaller sample sizes, we did not include interaction terms in these models. Row 3 therefore acts as a reference for the subsequent rows. As shown in the fourth row, our marginal odds ratios also did not differ substantially when we used 5 (versus 10) matched comparison drugs. As shown in rows five through seven, we also did not observe substantial differences in our marginal odds ratios using each supply chain issue database (FDA, ASHP, recalls, discontinuations) separately, compared to our main analysis using all databases together. Finally, we used nearest-neighbor propensity score methods to match drugs on month, formulation, drug class, provider-administered and WHO-essential-medicine status, years since market/generic availability, ≥5 manufacturers and ≥$5 million in sales at baseline. The results within our propensity-matched cohort are shown in the last row of each panel and did not differ substantially from our main results.

A. Meaningful Shortages

© 2024 Callaway Kim K et al. *JAMA Network Open.*

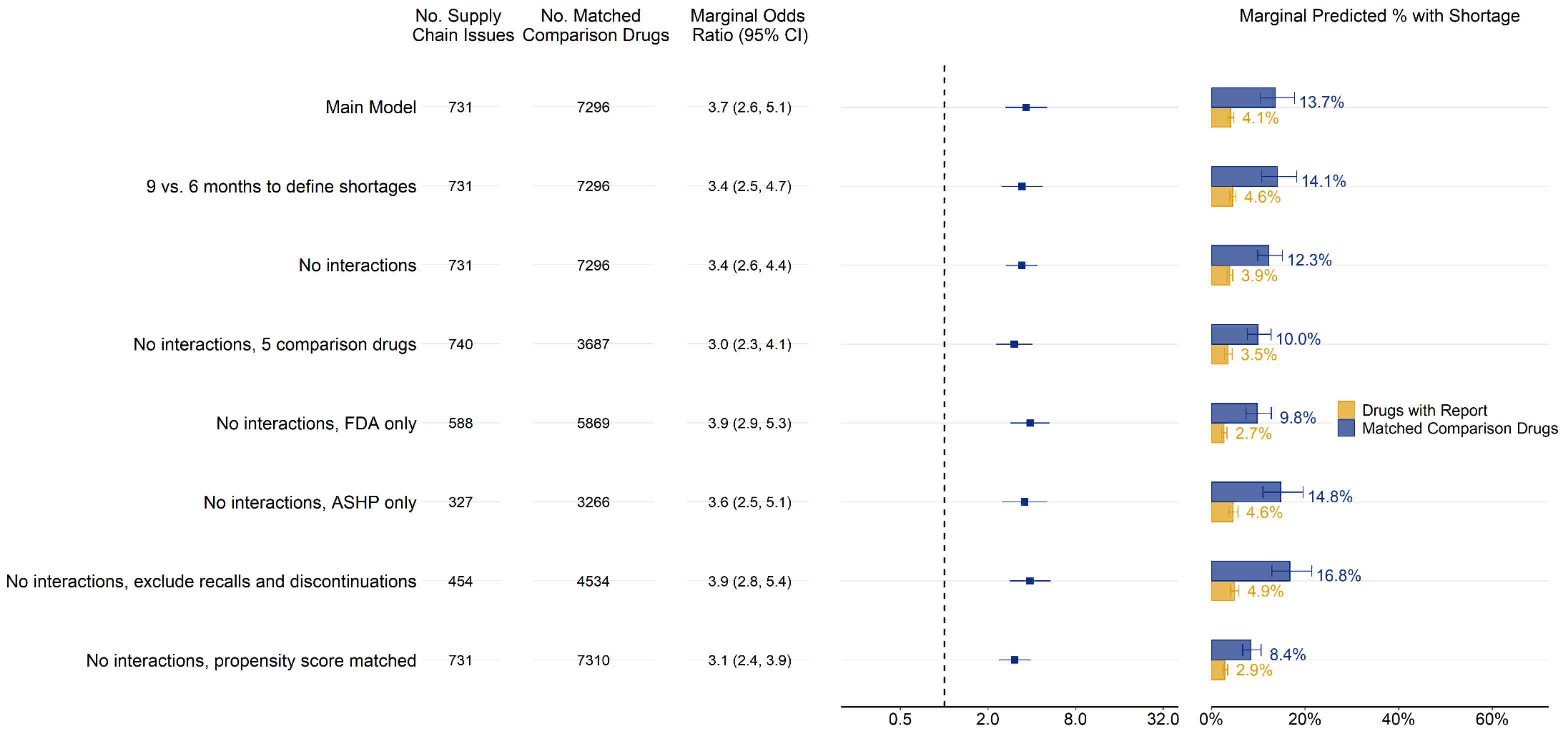

B. Severe Shortages

© 2024 Callaway Kim K et al. *JAMA Network Open.*

	No. Supply Chain Issues	No. Matched Comparison Drugs	Marginal Odds Ratio (95% CI)
Main Model	731	7296	6.3 (3.7,10.9)
9 vs. 6 months to define shortages	731	7296	6.2 (3.6,10.6)
No interactions	731	7296	4.9 (3.5, 6.9)
No interactions, 5 comparison drugs	740	3687	5.0 (3.1, 7.9)
No interactions, FDA only	588	5869	7.0 (4.6,10.8)
No interactions, ASHP only	327	3266	5.2 (3.0, 9.1)
No interactions, exclude recalls and discontinuations	454	4534	6.3 (3.9,10.4)
No interactions, propensity score matched	731	7310	4.4 (3.1, 6.0)

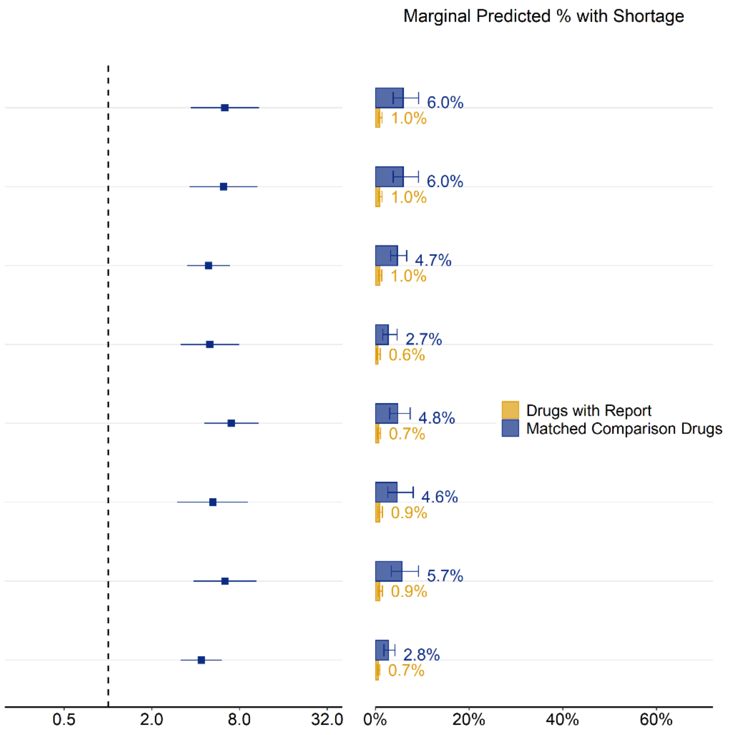

© 2024 Callaway Kim K et al. *JAMA Network Open.*

e-Table 7: Random Effects Logistic Regression Estimates, Odds of Meaningful (≥33%) and Severe (≥66%) Shortages for Supply Chain Issue Reports vs. Matched Comparison Drugs, Sensitivity Analysis Using 9 months to Define Shortages

e-Table 7 presents the full regression output for our sensitivity analysis using 9 (versus 6) months post-report to identify drug shortages. The results from our main model are shown in the first and fourth columns for reference. Odds ratios reflect the relationship between having the characteristic of interest and the outcome, conditional on other covariates and set-specific random effects. Due to a mathematical property of odds ratios called non-collapsibility, the conditional odds ratios in this table will be larger than the reported marginal odds ratios in e-Figure 7, which are effects estimated by "collapsing" or averaging over all other covariates and set-specific random effects.

Variable	Meaningful (≥33%) Shortage				Severe (≥66%) Shortage			
	Main Analysis Using 6 months to Define Shortages		Sensitivity Analysis Using 9 months to Define Shortages		Main Analysis Using 6 months to Define Shortages		Sensitivity Analysis Using 9 months to Define Shortages	
	OR (95% CI)[a]	p-val.	OR (95% CI)[a]	p-val.	OR (95% CI)[a]	p-val.	OR (95% CI)[a]	p-val.
Supply Issue Report	4.53 (1.61,12.74)	0.004	4.49 (1.59,12.66)	0.005	17.01 (5.02,57.6)	<0.001	15.62 (4.6,53.06)	<0.001
Time period								
Jan. 2017 – Jan. 2020	Ref.	Ref.	Ref.	Ref.	Ref.	Ref.	Ref.	Ref.
Feb. 2020 – April 2020	2.55 (1.85,3.5)	<0.001	2.29 (1.65,3.17)	<0.001	1.23 (0.7,2.16)	0.47	1.28 (0.75,2.19)	0.37
May 2020 – Sept. 2021	0.9 (0.7,1.16)	0.42	0.76 (0.58,0.98)	0.033	1.21 (0.85,1.73)	0.30	1.06 (0.74,1.51)	0.76
Supply Issue * Time Period								
Jan. 2017 – Jan. 2020	Ref.	Ref.	Ref.	Ref.	Ref.	Ref.	Ref.	Ref.
Feb. 2020 – April 2020	1.28 (0.51,3.22)	0.60	1.51 (0.6,3.77)	0.379	0.86 (0.18,4)	0.85	1.09 (0.26,4.5)	0.91
May 2020 – Sept. 2021	0.75 (0.4,1.42)	0.38	0.82 (0.44,1.54)	0.544	0.78 (0.36,1.7)	0.54	0.78 (0.36,1.69)	0.53
Formulation								
Oral	Ref.	Ref.	Ref.	Ref.	Ref.	Ref.	Ref.	Ref.
Parenteral	2.26 (1.72,2.97)	<0.001	2.33 (1.79,3.02)	<0.001	2.37 (1.59,3.53)	<0.001	2.28 (1.57,3.33)	<0.001
Ophthalmic or otic	1.37 (0.94,2.02)	0.11	1.42 (0.99,2.04)	0.058	0.91 (0.46,1.82)	0.79	0.95 (0.51,1.76)	0.86
Inhaled	1.22 (0.65,2.3)	0.54	1.15 (0.62,2.13)	0.663	1.61 (0.66,3.92)	0.30	1.53 (0.66,3.55)	0.32
Supply Issue * Formulation								
Oral	Ref.	Ref.	Ref.	Ref.	Ref.	Ref.	Ref.	Ref.
Parenteral	0.35 (0.16,0.76)	0.008	0.36 (0.16,0.77)	0.009	0.16 (0.06,0.46)	0.001	0.18 (0.07,0.5)	0.001
Ophthalmic or otic	1.52 (0.46,5.01)	0.49	1.35 (0.41,4.51)	0.622	1.02 (0.22,4.69)	0.975	0.87 (0.19,4.03)	0.86
Inhaled	0.46 (0.07,3.04)	0.42	0.45 (0.07,2.95)	0.408	0.38 (0.04,3.4)	0.39	0.33 (0.04,3.07)	0.33
≥ 5 manufacturers	0.34 (0.24,0.48)	<0.001	0.33 (0.24,0.46)	<0.001	0.19 (0.09,0.41)	<0.001	0.16 (0.07,0.36)	<0.001
Supply Issue * ≥ 5 manu.	0.59 (0.26,1.35)	0.21	0.5 (0.22,1.13)	0.097	0.72 (0.17,2.96)	0.65	0.72 (0.17,2.96)	0.64
WHO essential medicine	0.98 (0.78,1.22)	0.83	0.95 (0.76,1.18)	0.626	0.88 (0.61,1.27)	0.50	0.86 (0.61,1.21)	0.38
Supply Issue* WHO essential med.	0.49 (0.27,0.88)	0.02	0.55 (0.31,0.98)	0.044	0.32 (0.13,0.74)	0.008	0.32 (0.14,0.74)	0.008

© 2024 Callaway Kim K et al. *JAMA Network Open.*

Provider-administered drug	0.48 (0.36,0.64)	<0.001	0.46 (0.35,0.61)	<0.001	0.49 (0.32,0.75)	0.001	0.43 (0.28,0.66)	<0.001
Supply Issue * Prov.-administered drug	2.14 (0.96,4.74)	0.06	1.97 (0.9,4.31)	0.091	3.23 (1.09,9.62)	0.04	2.94 (1.01,8.52)	0.05
Marketing Category								
Generic (ANDA)	Ref.	Ref.	Ref.	Ref.	Ref.	Ref.	Ref.	Ref.
Brand-name (NDA)	0.92 (0.71,1.2)	0.55	1.01 (0.79,1.29)	0.952	0.95 (0.65,1.4)	0.80	1.14 (0.8,1.64)	0.46
Other or unknown	0.92 (0.66,1.29)	0.64	0.87 (0.63,1.2)	0.405	1.1 (0.66,1.83)	0.71	1.19 (0.73,1.94)	0.48
Supply Issue * Marketing Category								
Generic (ANDA)	Ref.	Ref.	Ref.	Ref.	Ref.	Ref.	Ref.	Ref.
Brand-name (NDA)	3.32 (1.54,7.15)	0.002	3.15 (1.46,6.82)	0.004	2.78 (1.14,6.81)	0.03	2.52 (1.03,6.15)	0.04
Other or unknown	2.6 (0.99,6.8)	0.05	2.4 (0.92,6.25)	0.07	2.31 (0.65,8.17)	0.20	2.53 (0.74,8.68)	0.14
Baseline sales <5 mil. USD	3.65 (2.95,4.52)	<0.001	3.7 (3.02,4.54)	<0.001	6.92 (4.79,9.99)	<0.001	7.35 (5.2,10.39)	<0.001
Supply Issue * Baseline sales <5 million USD	1.14 (0.62,2.08)	0.68	1 (0.56,1.81)	0.99	0.54 (0.24,1.26)	0.16	0.52 (0.23,1.19)	0.12
Age Category								
<5 years	Ref.	Ref.	Ref.	Ref.	Ref.	Ref.	Ref.	Ref.
5-9 years	0.86 (0.62,1.19)	0.37	1.03 (0.75,1.41)	0.85	0.8 (0.5,1.29)	0.36	0.83 (0.53,1.3)	0.42
10-19 years	0.82 (0.59,1.13)	0.23	0.89 (0.65,1.22)	0.48	0.61 (0.37,0.99)	0.05	0.64 (0.41,1.02)	0.06
≥20 years	0.85 (0.61,1.19)	0.35	0.98 (0.71,1.35)	0.90	0.63 (0.38,1.03)	0.07	0.71 (0.44,1.13)	0.15
Supply Issue * Age Category								
< 5 years	Ref.	Ref.	Ref.	Ref.	Ref.	Ref.	Ref.	Ref.
5-9 years	1.13 (0.39,3.34)	0.82	0.98 (0.33,2.91)	0.97	1.04 (0.31,3.51)	0.95	1.04 (0.3,3.58)	0.95
10-19 years	0.96 (0.34,2.66)	0.93	1.03 (0.37,2.89)	0.95	0.74 (0.22,2.48)	0.63	0.85 (0.25,2.85)	0.80
≥20 years	0.93 (0.34,2.54)	0.89	0.94 (0.34,2.6)	0.91	0.72 (0.22,2.32)	0.58	0.75 (0.23,2.43)	0.63
Intercept	0.04 (0.03,0.06)	<0.001	0.04 (0.03,0.06)	<0.001	0.01 (0.01,0.02)	<0.001	0.01 (0.01,0.02)	<0.001
Dist. of Set-Specific Random Effects								
ICC (SE)	0.07 (0.03)		0.1 (0.02)		0.06 (0.05)		0.11 (0.04)	

Abbreviations: OR = odds ratio; p-val. = p-value; manu. = manufacturers; mil. = million, Ref. = reference category; Prov. = provider; Dist. = distribution; se=standard error

a. Odds ratios reflect the relationship between having the characteristic of interest and the outcome, conditional on other covariates and set-specific random effects. Due to a mathematical property of odds ratios called non-collapsibility, the conditional odds ratios in this table will be larger than the reported marginal odds ratios in e-Figure 7, which are effects estimated by "collapsing" or averaging over all other covariates and set-specific random effects.

© 2024 Callaway Kim K et al. *JAMA Network Open.*

e-Table 8: Random Effects Logistic Regression Estimates, Odds of Meaningful (≥33%) Shortages for Supply Chain Issue Reports vs. Matched Comparison Drugs, Sensitivity Analyses Using Different Inclusion Criteria

e-Table 8 presents the full regression output for our sensitivity analyses looking at the odds of meaningful (≥33%) shortages, using 5 (versus 10) matched comparison drugs, restricting to FDA reports only, restricting to ASHP reports only, and excluding recalls and discontinuations. Our results from our main model are shown in the first column for reference. Odds ratios reflect the relationship between having the characteristic of interest and the outcome, conditional on other covariates and set-specific random effects. Due to a mathematical property of odds ratios called non-collapsibility, the conditional odds ratios in this table will be larger than the reported marginal odds ratios in e-Figure 7, which are effects estimated by "collapsing" or averaging over all other covariates and set-specific random effects.

Variable	Main Analysis Using All Reports and 10 matched comparison drugs		Sensitivity Analysis Using All Reports and 5 matched comparison drugs		Sensitivity Analysis Using only FDA website		Sensitivity Analysis Using only ASHP website		Sensitivity Analysis Excluding Recalls and Discontinuations	
	OR (95% CI)[a]	p-val.	OR (95% CI)[a]	p-val.	OR (95% CI)[a]	p-val.	OR (95% CI)[a]	p-val.	OR (95% CI)[a]	p-val.
Supply Issue Report	3.41 (2.65,4.39)	<0.001	3.03 (2.27,4.05)	<0.001	3.9 (2.86,5.33)	<0.001	3.58 (2.51,5.13)	<0.001	3.89 (2.81,5.37)	<0.001
Time period										
Jan. 2017 – Jan. 2020	Ref.	Ref.	Ref.	Ref.	Ref.	Ref.	Ref.	Ref.	Ref.	Ref.
Feb. 2020 – April 2020	2.55 (1.88,3.47)	<0.001	2.11 (1.39,3.21)	<0.001	2.14 (1.45,3.16)	<0.001	1.84 (1.14,2.96)	0.01	2.32 (1.52,3.56)	<0.001
May 2020 – Sept. 2021	0.89 (0.71,1.13)	0.36	1.01 (0.75,1.38)	0.93	0.8 (0.58,1.09)	0.16	0.66 (0.45,0.97)	0.03	0.66 (0.46,0.95)	0.025
Formulation										
Oral	Ref.	Ref.	Ref.	Ref.	Ref.	Ref.	Ref.	Ref.	Ref.	Ref.
Parenteral	1.97 (1.52,2.54)	<0.001	1.49 (1.03,2.16)	0.04	1.82 (1.31,2.52)	<0.001	2.01 (1.39,2.91)	<0.001	1.91 (1.35,2.7)	<0.001
Ophthalmic or otic	1.41 (0.99,2.02)	0.06	1.76 (1.12,2.77)	0.01	1.12 (0.7,1.78)	0.65	1.89 (1.15,3.13)	0.01	1.72 (1.08,2.75)	0.023
Inhaled	1.14 (0.64,2.06)	0.65	1.85 (0.91,3.75)	0.09	0.98 (0.44,2.16)	0.95	0.87 (0.34,2.26)	0.78	0.96 (0.42,2.2)	0.93
≥ 5 manu. at baseline	0.3 (0.22,0.41)	<0.001	0.38 (0.25,0.56)	<0.001	0.27 (0.18,0.4)	<0.001	0.31 (0.19,0.49)	<0.001	0.39 (0.26,0.59)	<0.001
WHO essential medicine	0.88 (0.72,1.08)	0.23	0.87 (0.66,1.15)	0.34	0.77 (0.59,1.01)	0.06	1.19 (0.89,1.58)	0.25	1.18 (0.9,1.55)	0.23

© 2024 Callaway Kim K et al. *JAMA Network Open.*

Prov.-administered drug	0.51 (0.39,0.67)	<0.001	0.81 (0.56,1.18)	0.27	0.55 (0.39,0.78)	0.001	0.51 (0.35,0.74)	<0.001	0.52 (0.36,0.74)	<0.001
Marketing Category										
Generic (ANDA)	Ref.	Ref.	Ref.	Ref.	Ref.	Ref.	Ref.	Ref.	Ref.	Ref.
Brand-name (NDA)	1.05 (0.83,1.33)	0.69	1.05 (0.75,1.48)	0.76	0.93 (0.69,1.27)	0.66	1.08 (0.76,1.53)	0.66	1.14 (0.82,1.58)	0.43
Other or unknown	1.06 (0.77,1.44)	0.73	1.43 (0.92,2.21)	0.11	0.77 (0.51,1.17)	0.22	1.03 (0.67,1.6)	0.89	0.95 (0.63,1.45)	0.82
Baseline sales <5 mil. USD	3.85 (3.16,4.7)	<0.001	3.97 (2.99,5.26)	<0.001	2.86 (2.23,3.68)	<0.001	2.58 (1.95,3.43)	<0.001	2.49 (1.91,3.25)	<0.001
Age Category										
<5 years	Ref.	Ref.	Ref.	Ref.	Ref.	Ref.	Ref.	Ref.	Ref.	Ref.
5-9 years	0.84 (0.62,1.15)	0.28	0.79 (0.51,1.23)	0.30	0.8 (0.54,1.2)	0.28	0.67 (0.42,1.05)	0.08	0.72 (0.47,1.11)	0.13
10-19 years	0.8 (0.59,1.09)	0.16	0.7 (0.46,1.08)	0.12	0.93 (0.63,1.36)	0.70	0.82 (0.53,1.25)	0.35	0.88 (0.58,1.32)	0.54
≥20 years	0.83 (0.61,1.13)	0.24	0.87 (0.57,1.34)	0.53	0.85 (0.57,1.27)	0.43	0.77 (0.49,1.2)	0.24	0.86 (0.56,1.3)	0.47
Intercept	0.04 (0.03,0.06)	<0.001	0.03 (0.02,0.06)	<0.001	0.04 (0.02,0.06)	<0.001	0.05 (0.03,0.09)	<0.001	0.05 (0.03,0.08)	<0.001
Distributions of Set-Specific Random Effects										
ICC (SE)	0.07 (0.03)		0.07 (0.05)		0.13 (0.03)		0.06 (0.04)		0.06 (0.03)	

Abbreviations: OR = odds ratio; p-val. = p-value; Ref.= reference category; se=standard error

a. Odds ratios reflect the relationship between having the characteristic of interest and the outcome, conditional on other covariates and set-specific random effects. Due to a mathematical property of odds ratios called non-collapsibility, the conditional odds ratios in this table will be larger than the reported marginal odds ratios in e-Figure 7, which are effects estimated by “collapsing” or averaging over all other covariates and set-specific random effects.

© 2024 Callaway Kim K et al. *JAMA Network Open.*

e-Table 9: Random Effects Logistic Regression Estimates, Odds of Severe (≥66%) Shortages in MIDAS Supply for Supply Chain Issue Reports vs. Matched Comparison Drugs, Sensitivity Analyses Using Different Inclusion Criteria

e-Table 9 presents the full regression output for our sensitivity analyses looking at the odds of severe (≥66%) shortages, using 5 (versus 10) matched comparison drugs, restricting to FDA reports only, restricting to ASHP reports only, and excluding recalls and discontinuations. The results from our main model are shown in the first column for reference. Odds ratios reflect the relationship between having the characteristic of interest and the outcome, conditional on other covariates and set-specific random effects. Due to a mathematical property of odds ratios called non-collapsibility, the conditional odds ratios in this table will be larger than the reported marginal odds ratios in e-Figure 7, which are effects estimated by "collapsing" or averaging over all other covariates and set-specific random effects.

Variable	Main Analysis Using All Reports and 10 matched comparison drugs		Sensitivity Analysis Using All Reports and 5 matched comparison drugs		Sensitivity Analysis Using only FDA website		Sensitivity Analysis Using only ASHP website		Sensitivity Analysis Excluding Recalls and Discontinuations	
	OR (95% CI) [a]	p-val.	OR (95% CI) [a]	p-val.	OR (95% CI) [a]	p-val.	OR (95% CI) [a]	p-val.	OR (95% CI) [a]	p-val.
Supply Issue Report	4.91 (3.5,6.88)	<0.001	5 (3.15,7.95)	<0.001	7.03 (4.56,10.84)	<0.001	5.24 (3,9.15)	<0.001	6.35 (3.87,10.42)	<0.001
Time period										
Jan. 2017 – Jan. 2020	Ref.	Ref.	Ref.	Ref.	Ref.	Ref.	Ref.	Ref.	Ref.	Ref.
Feb. 2020 – April 2020	1.18 (0.7,2.01)	0.53	1.32 (0.58,3.02)	0.50	1.16 (0.61,2.22)	0.65	0.42 (0.1,1.81)	0.25	0.89 (0.33,2.41)	0.82
May 2020 – Sept. 2021	1.19 (0.87,1.64)	0.28	0.89 (0.52,1.52)	0.67	0.85 (0.53,1.35)	0.48	1.35 (0.78,2.35)	0.29	1.19 (0.69,2.05)	0.53
Formulation										
Oral	Ref.	Ref.	Ref.	Ref.	Ref.	Ref.	Ref.	Ref.	Ref.	Ref.
Parenteral	1.76 (1.22,2.54)	0.002	1.14 (0.61,2.13)	0.68	1 (0.57,1.76)	0.999	1.74 (0.93,3.26)	0.09	1.37 (0.76,2.48)	0.30
Ophthalmic or otic	0.93 (0.51,1.7)	0.82	0.87 (0.36,2.09)	0.76	0.93 (0.43,2.02)	0.85	1 (0.34,2.98)	0.999	0.91 (0.34,2.44)	0.85
Inhaled	1.42 (0.64,3.15)	0.38	2.08 (0.71,6.11)	0.18	0.62 (0.14,2.71)	0.53	1.38 (0.31,6.1)	0.67	1.31 (0.37,4.61)	0.68
≥ 5 manufacturers at baseline	0.18 (0.1,0.35)	<0.001	0.17 (0.07,0.41)	<0.001	0.17 (0.08,0.37)	<0.001	0.23 (0.09,0.61)	0.003	0.26 (0.11,0.59)	0.002
WHO essential medicine	0.7 (0.5,0.98)	0.036	0.73 (0.44,1.18)	0.20	0.61 (0.38,0.98)	0.04	0.96 (0.57,1.64)	0.89	1.2 (0.74,1.93)	0.47

© 2024 Callaway Kim K et al. *JAMA Network Open.*

Provider-administered drug	0.56 (0.38,0.83)	0.004	0.94 (0.5,1.78)	0.85	0.93 (0.51,1.7)	0.81	0.55 (0.29,1.03)	0.06	0.54 (0.29,0.99)	0.047
Marketing Category										
Generic (ANDA)	Ref.	Ref.	Ref.	Ref.	Ref.	Ref.	Ref.	Ref.	Ref.	Ref.
Brand-name (NDA)	1.11 (0.79,1.57)	0.54	1.26 (0.73,2.18)	0.40	0.83 (0.5,1.37)	0.46	0.68 (0.36,1.26)	0.22	0.7 (0.39,1.27)	0.24
Other or unknown	1.27 (0.8,2.01)	0.31	1.23 (0.58,2.64)	0.59	0.84 (0.42,1.68)	0.61	1 (0.47,2.15)	0.998	1.13 (0.55,2.32)	0.74
Baseline sales <5 million USD	6.45 (4.67,8.91)	<0.001	4.01 (2.48,6.48)	<0.001	3.24 (2.11,4.97)	<0.001	3.54 (2.13,5.87)	<0.001	3.55 (2.2,5.72)	<0.001
Age Category										
<5 years	Ref.	Ref.	Ref.	Ref.	Ref.	Ref.	Ref.	Ref.	Ref.	Ref.
5-9 years	0.76 (0.5,1.16)	0.21	0.51 (0.25,1.06)	0.07	0.53 (0.28,1)	0.05	0.47 (0.22,1.01)	0.05	0.43 (0.21,0.88)	0.02
10-19 years	0.55 (0.36,0.86)	0.008	0.63 (0.32,1.25)	0.19	0.56 (0.3,1.03)	0.06	0.47 (0.23,0.98)	0.04	0.48 (0.24,0.95)	0.04
≥20 years	0.57 (0.36,0.89)	0.01	0.7 (0.35,1.38)	0.31	0.62 (0.33,1.14)	0.12	0.35 (0.16,0.76)	0.007	0.36 (0.18,0.74)	0.005
Intercept	0.01 (0.01,0.02)	<0.001	0.01 (0,0.02)	<0.001	0.02 (0.01,0.04)	<0.001	0.02 (0.01,0.05)	<0.001	0.02 (0.01,0.05)	<0.001
Distributions of Set-Specific Random Effects										
ICC (SE)	0.07 (0.05)		0.25 (0.09)		0.06 (0.08)		0.09 (0.09)		0.12 (0.08)	

Abbreviations: OR = odds ratio; p-val. = p-value; Ref.= reference category; se=standard error

a. Odds ratios reflect the relationship between having the characteristic of interest and the outcome, conditional on other covariates and set-specific random effects. Due to a mathematical property of odds ratios called non-collapsibility, the conditional odds ratios in this table will be larger than the reported marginal odds ratios in e-Figure 7, which are effects estimated by "collapsing" or averaging over all other covariates and set-specific random effects.

© 2024 Callaway Kim K et al. *JAMA Network Open.*

e-Table 10: Characteristics of Supply Chain Issue Reports vs. Matched Comparison Drugs, 2017-2021, Sensitivity Analysis Among Propensity-Score-Matched Cohort

e-Table 10 present descriptive statistics for our sensitivity cohort for which supply chain issue reports were nearest neighbor matched to up to 10 comparison drugs with no concurrent report. Characteristics of drugs in our main cohort are shown in the first two columns for reference. Matching was conducted using propensity scores calculated from logistic regression models predicting supply chain issue report status (binary) from independent variables: calendar month (January 2017- September 2021), provider-administered status (binary), WHO essential medicines status (binary), ≥5 manufacturers in the 6 months prior to the report (binary), ≥$5 million sales in the 6 months prior to the report (binary), drug class (defined using WHO ATC3 category, as reported in MIDAS), drug formulation (oral, parenteral, ophthalmic/otic, or inhaled), and age group since initial FDA approval (<5 years, 5-9 years, 10-19 years, ≥20 years).

Variable	Main Cohort Matched Only on Supply Issue Month		Sensitivity Analysis Among Propensity-Matched Cohort	
	**Supply Chain Issue Reports (No. = 731)**	**Matched Comparison Drugs (No. = 7290)**	**Supply Chain Issue Reports (No. = 731)**	**Matched Comparison Drugs (No. = 7310)**
Time period, no. (%)				
January 2017 – January 2020	489 (67)		491 (67)	
February 2020 – April 2020	57 (8)		57 (8)	
May 2020 – September 2021	185 (25)		183 (25)	
Reporting agency[a], no. (%)				
FDA	588 (80)		585 (80)	
ASHP	327 (45)		318 (44)	
Type of Issue[a], no. (%)				
Shortage	363 (50)		355 (49)	
Recall	131 (18)		131 (18)	
Discontinuation	367 (50)		375 (51)	
Median (IQR) supply chain issue duration in months	3.7 (3,17.9)		3.25 (3,17.9)	
Duration category, no. (%)				
<6 months	401 (55)		400 (55)	
6-11 months	85 (12)		81 (11)	
12-23 months	109 (15)		106 (15)	
24-35 months	57 (8)		57 (8)	
≥36 months	79 (11)		78 (11)	
Reason for supply chain issue[b], no. (%)				
No or unspecified reason	264 (36)		273 (37)	
Manufacturing, packaging, or shipping issues	163 (22)		159 (22)	
Business Decision	108 (15)		108 (15)	
Discontinuation of the manufacture of the drug	73 (10)		71 (10)	
Impurities or lack of sterility	55 (8)		55 (8)	
Other Events	68 (9)		65 (9)	
Formulation, no. (%)				
Oral	403 (55)	4001 (55)	407 (56)	4324 (59)
Parenteral	286 (39)	2646 (36)	282 (39)	2672 (37)
Ophthalmic or otic	27 (4)	446 (6)	27 (4)	207 (3)
Inhaled	15 (2)	197 (3)	15 (2)	107 (1)

© 2024 Callaway Kim K et al. *JAMA Network Open.*

≥ 5 manufacturers at baseline[c], no. (%)	378 (52)	2225 (31)	382 (52)	3725 (51)
WHO essential medicine, no. (%)	316 (43)	2199 (30)	316 (43)	3215 (44)
Provider-administered drug, no. (%)	197 (27)	1730 (24)	192 (26)	2026 (28)
Marketing Category, no. (%)				
Generic available	616 (84)	4397 (60)	625 (85)	6315 (86)
Brand-name only	65 (9)	1812 (25)	65 (9)	578 (8)
Other or unknown	48 (7)	1040 (14)	41 (6)	417 (6)
Baseline sales <5 million USD [d], no. (%)	234 (32)	2152 (30)	227 (31)	2129 (29)
Median (IQR) drug age in years	20.4 (13.8,30.7)	14.5 (7.8,23.5)	20.3 (13.8,30.3)	20.5 (13.8,29.7)
Years since initial FDA approval, %				
<5 years	40 (5)	976 (13)	40 (5)	381 (5)
5-9 years	78 (11)	1468 (20)	77 (11)	682 (9)
10-19 years	238 (33)	2412 (33)	241 (33)	2383 (33)
≥20 years	375 (51)	2434 (33)	373 (51)	3864 (53)

Abbreviations: No. = number; IQR = interquartile range

a. Categories are not mutually exclusive since a single drug supply issue episode could include multiple events.

b. For episodes with multiple or conflicting reasons, we assigned the following priority ranking based on the typical temporal sequence of drug shortages: Shortage of raw ingredient > Manufacturing delay or Discontinuation > Increased demand. Other events included regulatory delays, shortages of active or inactive ingredients, demand increases, or other miscellaneous reasons.

c. Measured in MIDAS for the 6 months immediately prior to supply issue event initiation (baseline period).

© 2024 Callaway Kim K et al. *JAMA Network Open.*

e-Table 11: Random Effects Logistic Regression Estimates, Odds of Meaningful (≥33%) and Severe (≥66%) Shortages for Supply Chain Issue Reports vs. Matched Comparison Drugs, Sensitivity Analysis Among Propensity-Matched Cohort

e-Table 11 presents the full regression output for our sensitivity analysis among our propensity score matched cohort. The results from our main model are shown in the first and fourth columns for reference. Supply chain issue reports were nearest neighbor matched to up to 10 comparison drugs with no concurrent report. Matching was conducted using propensity scores calculated from logistic regression models predicting supply chain issue report status (binary) from independent variables: calendar month (January 2017- September 2021), provider-administered status (binary), WHO essential medicines status (binary), ≥5 manufacturers in the 6 months prior to the report (binary), ≥$5 million sales in the 6 months prior to the report (binary), drug class (defined using WHO ATC3 category, as reported in MIDAS), drug formulation (oral, parenteral, ophthalmic/otic, or inhaled), and age group since initial FDA approval (<5 years, 5-9 years, 10-19 years, ≥20 years). Odds ratios reflect the relationship between having the characteristic of interest and the outcome, conditional on other covariates and set-specific random effects. Due to a mathematical property of odds ratios called non-collapsibility, the conditional odds ratios in this table will be larger than the reported marginal odds ratios in e-Figure 7, which are effects estimated by "collapsing" or averaging over all other covariates and set-specific random effects.

Variable	Meaningful (≥33%) Shortage				Severe (≥66%) Shortage			
	Main Analysis Matching Only on Month		Sensitivity Analysis Among Propensity-Matched Cohort		Main Analysis Matching Only on Month		Sensitivity Analysis Among Propensity-Matched Cohort	
	OR (95% CI)[a]	p-val.	OR (95% CI)[a]	p-val.	OR (95% CI)[a]	p-val.	OR (95% CI)[a]	p-val.
Supply Issue Report	3.41 (2.65,4.39)	<0.001	3.06 (2.38,3.93)	<0.001	4.91 (3.5,6.88)	<0.001	4.36 (3.15,6.04)	<0.001
Time period								
Jan. 2017 – Jan. 2020	Ref.	Ref.	Ref.	Ref.	Ref.	Ref.	Ref.	Ref.
Feb. 2020 – April 2020	2.55 (1.88,3.47)	<0.001	3.08 (2.24,4.23)	<0.001	1.18 (0.7,2.01)	0.53	1.66 (0.95,2.91)	0.08
May 2020 – Sept. 2021	0.89 (0.71,1.13)	0.36	0.86 (0.68,1.08)	0.19	1.19 (0.87,1.64)	0.28	1.24 (0.91,1.69)	0.18
Formulation								
Oral	Ref.	Ref.	Ref.	Ref.	Ref.	Ref.	Ref.	Ref.
Parenteral	1.97 (1.52,2.54)	<0.001	1.89 (1.44,2.48)	<0.001	1.76 (1.22,2.54)	0.002	0.99 (0.65,1.49)	0.95
Ophthalmic or otic	1.41 (0.99,2.02)	0.06	2.14 (1.36,3.37)	0.001	0.93 (0.51,1.7)	0.82	1.07 (0.53,2.18)	0.85
Inhaled	1.14 (0.64,2.06)	0.65	0.83 (0.34,2.03)	0.68	1.42 (0.64,3.15)	0.38	0.86 (0.27,2.75)	0.81
≥ 5 manufacturers	0.3 (0.22,0.41)	<0.001	0.26 (0.19,0.36)	<0.001	0.18 (0.1,0.35)	<0.001	0.12 (0.06,0.24)	<0.001
WHO essential medicine	0.88 (0.72,1.08)	0.23	0.92 (0.75,1.13)	0.43	0.7 (0.5,0.98)	0.04	0.64 (0.46,0.89)	0.008
Provider-administered drug	0.51 (0.39,0.67)	<0.001	0.72 (0.55,0.95)	0.02	0.56 (0.38,0.83)	0.004	0.9 (0.58,1.39)	0.64
Marketing Category								
Generic (ANDA)	Ref.	Ref.	Ref.	Ref.	Ref.	Ref.	Ref.	Ref.
Brand-name (NDA)	1.05 (0.83,1.33)	0.69	1.23 (0.91,1.66)	0.18	1.11 (0.79,1.57)	0.54	1.38 (0.93,2.07)	0.11
Other or unknown	1.06 (0.77,1.44)	0.73	1.09 (0.71,1.67)	0.71	1.27 (0.8,2.01)	0.31	1.91 (1.03,3.56)	0.04
Baseline sales <5 mil. USD	3.85 (3.16,4.7)	<0.001	5.15 (4.07,6.51)	<0.001	6.45 (4.67,8.91)	<0.001	6.35 (4.28,9.42)	<0.001
Age Category								
<5 years	Ref.	Ref.	Ref.	Ref.	Ref.	Ref.	Ref.	Ref.
5-9 years	0.84 (0.62,1.15)	0.28	1.1 (0.72,1.68)	0.66	0.76 (0.5,1.16)	0.21	1.43 (0.82,2.5)	0.21

© 2024 Callaway Kim K et al. *JAMA Network Open.*

10-19 years	0.8 (0.59,1.09)	0.16	0.76 (0.51,1.14)	0.19	0.55 (0.36,0.86)	0.008	0.71 (0.4,1.26)	0.24
≥20 years	0.83 (0.61,1.13)	0.24	0.82 (0.55,1.22)	0.32	0.57 (0.36,0.89)	0.01	0.91 (0.53,1.57)	0.73
Intercept	0.04 (0.03,0.06)	<0.001	0.04 (0.02,0.06)	<0.001	0.01 (0.01,0.02)	<0.001	0.01 (0.01,0.03)	<0.001
Dist. of Set-Specific Random Effects								
ICC (SE)	0.07 (0.03)		0 (0.02)		0.07 (0.05)		0 (0)	

Abbreviations: OR = odds ratio; p-val. = p-value; manu. = manufacturers; mil. = million, Ref. = reference category; Prov. = provider; Dist. = distribution; se=standard error.

a. Odds ratios reflect the relationship between having the characteristic of interest and the outcome, conditional on other covariates and set-specific random effects. Due to a mathematical property of odds ratios called non-collapsibility, the conditional odds ratios in this table will be larger than the reported marginal odds ratios in e-Figure 7, which are effects estimated by ”collapsing” or averaging over all other covariates and set-specific random effects.

© 2024 Callaway Kim K et al. *JAMA Network Open.*
