# Supplementary material for: Drug Shortages Prior to and During the COVID-19 Pandemic
Source: JAMA Netw Open. 2024 Apr 5;7(4):e244246. doi: 10.1001/jamanetworkopen.2024.4246 (PMC10998160; doi:10.1001/jamanetworkopen.2024.4246)
Supplement: Supplement 2. — Data Sharing Statement [file jamanetwopen-e244246-s002.pdf]

Data Sharing Statement

Data
Data available: No

Additional Information
Explanation for why data not available: The data is proprietary. However, we will provide a data dictionary and additional analysis upon request.
